# Formal Manual Fecal Sludge Emptying and Transport Services: Cost Analysis of 23 Service Providers in Malawi and Uganda

**DOI:** 10.1021/acs.est.6c01385

**Published:** 2026-05-20

**Authors:** Jonathan D. T. Wilcox, Carlos E. V. Batarda, Julita C. Chinseu, Yvonne S. Lugali, Jamie K. Bartram, Barbara E. Evans

**Affiliations:** † 4468 University of Leeds, Leeds LS2 9JT, United Kingdom; ‡ WaterAid, Sixth Floor, 20 Canada Square, London E14 5NN, United Kingdom; § Water For People Uganda, Plot 15b Kitanta Close, Off Yusuf Lule Road, PO Box 4120, Kampala 25601, Uganda; ∥ Water For People Malawi, PO Box 1207, Blantyre 312200, Malawi

**Keywords:** faecal sludge management, desludging, whole-life costing, safely managed sanitation, pit latrine emptying

## Abstract

In East and Southern African cities, pit latrines are the most common household sanitation system and often require manual emptying. City authorities in the region have begun to formalize service providers, and, along with service providers, require cost data and analysis to optimize service delivery and reduce costs to minimize public funding contributions toward a large funding gap. We analyzed financial and operational data from 23 formal service providers and 260 households in Malawi and Uganda. Total Annualized Cost per Household (TACH) was calculated to understand unit costs and cost structures, and scenario modeling was used to analyze cost drivers. Manual emptying is higher TACH than mechanical emptying at full capacity utilization because of higher labor costs. TACH varies between households due to different annualized sludge emptying rates. Service providers report operating below full capacity, increasing TACH and not justifying vehicle ownership over rental. Households prefer low-volume and low-price emptying to manage budgets, but this increases TACH unless service providers coordinate to maximize capacity. Options are discussed for city authorities and service providers to minimize TACH and improve vertical equity between emptying methods and horizontal equity between households, including replacing direct user-payments with frequently collected citywide payments and managed emptying services.

## Graphical abstract

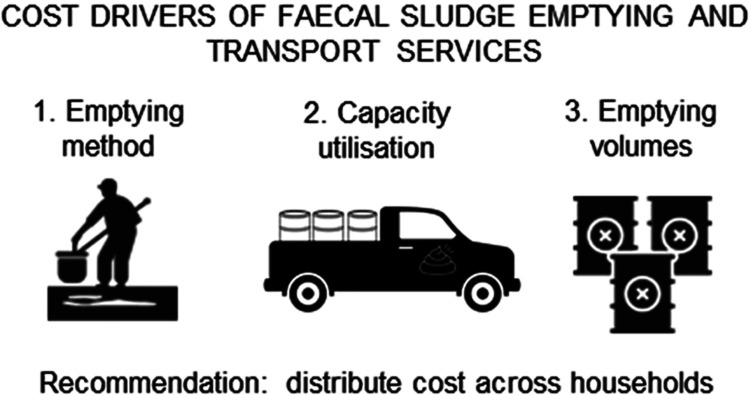

## Introduction

Pit latrines are the most common household sanitation system in cities in Sub-Saharan Africa^1^ particularly in informal settlements.^2^ As cities become more densely populated, there is often no space to replace latrines when they are full. To protect public and environmental health, pits must be emptied and the sludge safely transported to an appropriate disposal or treatment site.^3^ Growing urban populations and the improved access to household sanitations means that the amount of sludge requiring emptying is fast increasing.^4^

In many cities private sector service providers empty septic tanks and pit latrines using exhauster trucks, but exhauster trucks may be unable to access pits in dense settlements without roads, handle trash and to remove thick sludge.^5–7^ Manual emptying (using either a manually operated pump such as the Gulper, or buckets and rope)^8^ is therefore required, and is typically done informally and the sludge not safely disposed.^9^ In response, several cities have formalized manual emptying service providers. Licenses from the relevant authorities^10^ generally require the adoption of more hygienic technologies (such as the Gulper^11,12^ ) and the transport of sludge in barrels to a treatment or a safe disposal site. Formalisation incurs additional safety, licensing, and transportation costs;^13^ manual emptying is slower;^14^ and some systems cannot be emptied using methods accepted by licensing authorities.^5,7^ Furthermore, the benefits are not private and it does not substantially increase willingness to pay, which increases the large funding gap.^15,16^ Households paying for emptying services prefer to reduce individual payments to help manage budgets.^9^ Service providers have responded by offering volumetric tariffs and removing smaller sludge volumes.^7,10,17^ As sanitation is a public good,^18^ market-based systems will not provide universal services without additional incentives^19^ and since sanitation is also a merit good,^18^ cities are increasingly revisiting how to increase coverage of formal services by adopting a public service approach.^20^

City authorities need more cost data and analysis to inform financial planning^21^ to support both private sector participation and public service delivery e.g., cost underestimation has caused challenges for subsidy design.^22^ Previous peer reviewed studies have found that costs could be lowered by clustering jobs at the neighborhood level,^23,24^ the additional costs of formalization are significant,^13^ and operating systems below design capacity increases average costs.^25^ But these studies analyzed individual formal manual emptying service providers, not allowing for cross-comparison to improve the reliability and generalizability of findings.

In this study we analyze empirical operational and financial data from a household and a service provider survey in Blantyre, Malawi and Kampala, Uganda produced by Water For People (an international non-governmental organization headquartered in the USA). Data was produced from service providers who have previously received support from Water For People, either directly or indirectly through market-system development.^26^ A whole-life cost accounting methodology developed specifically for urban sanitation and city authorities^8^ is used to organize cost data to understand service providers’ unit costs and cost structures, and to calculate the Total Annualized Cost per Household (TACH). TACH was developed and is used in this study because it represents the full financial cost of delivering a component of the sanitation service chain (emptying and transport in this study) and is expressed in a unit (annualized cost per household) that is most useful to city authorities by aligning with annual budgeting, enabling comparison to other public services. TACH is calculated for a typical small-scale service provider and scenario modeling conducted on (1) household emptying characteristics (e.g., due to different emptying volumes and intervals), (2) operating scale (emptying jobs per year) and capacity utilization (fully using equipment), and (3) emptying volume per job. Our overall objective is to support both city authorities and service providers with financial and operational planning toward achieving citywide safely managed sanitation.

## Materials and Methods

### Data Familiarization

In this study we used empirical data from a household survey and a service provider survey conducted by phone by Water For People and Le Fil Consulting between April and December 2021 in Blantyre, Malawi and Kampala, Uganda: 238 households (140 from Blantyre and 98 from Kampala) and 23 service providers (10 from Blantyre and 13 from Kampala) responded. Water For People have supported households, and emptying and transport service providers in both cities to both improve and increase safely managed sanitation coverage.^4,12^ The households surveyed were users of the supported service providers. The data sets were produced and analyzed to evaluate Water For People’s support in both cities.^10^ Informed consent was obtained for Water For People to share anonymized survey data with other researchers for analysis.

For this study, both data sets were treated as secondary because analysis was planned after data production.^27^ Data familiarization was conducted using qualitative cross-case comparative analysis^28^ which consisted of comparing the service providers by visualizing the data: either ordering the data and using bar charts, or comparing two continuous variables using scatter plots. Figures from data familiarization are included in Supporting Information. Data familiarization was combined with a preliminary literature review to identify analytical limitations and opportunities, potential research questions and analytical methods.^29^ Discussion with Water For People staff reduced the risk arising from unfamiliarity with the data production processes.

### Data Analysis

#### Definitions

The term “job” is used to refer to an emptying event consisting of a single transaction between a household and a service provider. A job may consist of multiple barrels emptied and trips by vehicle to the disposal or treatment site. “Capacity utilisation” refers to the proportion of the maximum capacity for that emptying and transport method i.e., full capacity utilization is two jobs per day for manual emptying with transport using a pickup truck. “Operating scale” refers to the number of jobs completed per day (or per year). The term “service provider” refers to the businesses providing the emptying service, and “emptier” refers to a laborer who removes sludge. “Emptying interval” is the time duration between each emptying job for an individual containment system. “Working day” refers to Monday through Saturday i.e., 6 days per week.

#### Cost Estimating

Unit costs and operating parameters were reviewed and organized using a cost category pro-forma into direct and indirect costs, and capital and operational expenditure (CAPEX and OPEX). Unit costs and quantities were used to calculate TACH based on an equivalent annual cost (EAC) approach: see eq 1. The full “industrial cost” was estimated, including both direct and indirect costs of operating the service, plus administrative costs, financing costs and taxation. Cost and revenue streams between service providers along the sanitation service chain were excluded e.g., dumping fees at a treatment plant, to allow costs from other segments of the sanitation value chain to be summed to produce a complete cost estimate for safely managed sanitation. Cost data were normalized between countries and years using purchasing power parity and inflation data to 2020 international dollars (Int$_2020_),^30^ and annualized based on a 5% social discount rate.^8^ Sensitivity analysis was not performed on the social discount rate because CAPEX is a small proportion of TACH for emptying and transport services.

1 $$\mathrm{EAC}=\frac{k_{n}\cdot \sum_{t=0}^{T}\frac{{\mathrm{COST}}_{t}\cdot k_{n}}{{\left(1+k_{n}\right)}^{t}}}{{1-\left(1+k_{n}\right)}^{-T}}$$

Modeling was based on a service provider consisting of a single team operating at the assumed full capacity for the different emptying and transport methods reported in Water For People’s study:^10^ one job per working day for manual emptying and transport using a tricycle; two jobs per working day for manual emptying and transport using a pickup; and four jobs per working day for mechanical emptying and transport using a pickup truck. These maximum capacities were corroborated by other studies from the same or similar contexts.^6,7,14^ Median values for each input parameter were used to minimize the influence of outliers in the data set and to avoid aggregating uncertainty.

Analytical cost estimating was used to calculate TACH rather than parametric cost estimating. In simple terms, analytical cost estimating is a bottom-up approach comparing individual unit costs and quantities across service providers, and parametric cost estimating is a top-down approach comparing the total cost and operating variables between service providers. Analytical estimating was preferred because of gaps in the survey data at the service provider level (i.e., not all service providers reported their full cost) and also to minimize the influence of input parameter outliers on calculating individual service provider TACH. All modeling assumed all variables to be independent.

Barrels, hooks, Gulpers, personal protective equipment (PPE) and emptying water (which is used to fluidize sludge to ease emptying) were grouped into equipment. Equipment costs were modeled by multiplying the number of units per emptier, the number of emptiers per team and the unit cost per emptier, and annualized assuming a five year lifetime for equipment, two years for PPE, and no financing costs. Emptying water showed no relationship with operating scale, although intuitively water consumption would be expected to increase with emptying volume, and was modeled as a fixed cost to be consistent with other variables that are modeled as per observed trend in the data. Emptiers’ wages were modeled by multiplying the total wage (for all emptiers combined) per barrel emptied, the average number of barrels per job and the total number of jobs per year. Calculating emptiers’ wages was based on per barrel wages normalized for changes in vehicle barrel capacity and different emptying volumes. Vehicle capital and finance costs were modeled as a fixed cost and annualized based on an assumed 10 year lifetime and financed by a three year, 22% interest per year loan and a 10% deposit (suggested by Water For People staff based on experience). Few service providers reported vehicle capital cost and financing terms, so these specifically were confirmed with Water For People staff. Fuel costs were modeled by multiplying fuel cost per trip, average number of trips per job and total jobs per year. Average number of trips per job was determined by the vehicle barrel capacity and the number of barrels emptied per job. Referral costs were modeled by multiplying the referral payment per barrel emptied, proportion of customers referred by brokers, and total emptying jobs. Service providers paying brokers per job or a salary were normalized per barrel.

Indirect cost were grouped into marketing; management (nonemptying) salaries; office, supplies and utilities; licenses; and taxes. Service providers reported withholding tax, value-added tax, and corporate taxes related to revenue. For simplicity, total tax was modeled as a percentage of total costs. All other indirect costs were modeled as fixed costs as no relationship was observed with operating scale.

The total number of households served per year was modeled as total jobs per year multiplied by average number of households sharing a latrine and the average interval between household emptying jobs.^8^ The total jobs per year was modeled as the number of teams multiplied by the number of working days and the average number of jobs per working day per team. The average number of households sharing a latrine was calculated using the proportion of households sharing and the average number of households sharing; this accounts for systems which were shared by multiple households. Emptying interval was reported in the household survey as every month, a few times per year, once a year, or every few years; and was quantified respectively as 0.08, 0.33, 1, and 2.5 years. The interval between emptying jobs was assumed to be directly related to the sludge volume per job. The average number of households sharing a latrine was assumed to be four.^31^

### Scenario Modeling

To understand TACH cost drivers, input parameters were varied in three modeling scenarios.

Variation in TACH between households was modeled by varying three factors reported by 52 households in the household survey: emptying volume, emptying interval, and whether households reported sharing sanitation systems. The annualized household sludge emptying rate (m^3^ per household per year) was calculated for each household to normalize emptying volumes by emptying interval. The Total Annualized Volumetric Cost was calculated by dividing TACH by the annualized household sludge emptying rate.

The relationship between operating scale and capacity utilization, and TACH was modeled by varying the total number of manual emptying jobs completed between approximately the 10%ile (100 jobs per year) and 90%ile values (1500 jobs per year) reported by service providers. Vehicle ownership and rental costs at different operating scales and capacity utilization were compared by assuming that vehicle ownership was a fixed cost (up to full capacity utilization) and that vehicle rental was a variable cost (per job). The *financial tipping point* was calculated as the operating scale at which the annual loan repayment for the duration of the loan (three years) was equal to the annual rental cost i.e., when using a loan to purchase a vehicle is lower cost during the loan repayment period than renting. The *economic tipping point* was calculated as the operating scale at which the annualized loan repayment for the duration of the asset (ten years) was equal to the annual rental cost i.e., when the using a loan to purchase a vehicle is lower cost throughout the asset lifetime than renting.

The impact on TACH of reducing emptying volumes per job was modeled by assuming that the annualized household sludge emptying rate remains constant for households, i.e., emptying volume per job is directly proportional to emptying interval. Two scenarios were modeled: constant jobs per year where the service provider completes two jobs per working day with a variable total annual emptying volume; and constant volume per year where the service provider completes a variable number of jobs per year with a constant total annual emptying volume (equivalent to two 11 barrel jobs per working day) with multiple jobs per day or idle days as required to maintain a constant total annual emptying volume.

## Results and Discussion

### Service Providers Have Diverse Cost Structures and Unit Costs

The service provider data revealed a diversity in operating models and associated cost structures. The open market in both cities allows service providers to align with different customer groups, using suitable technology and emptying methods:^10^ smaller volumes with tricycles, medium volumes with pickups, and where possible with exhauster trucks.

Table 1 summarizes the different cost structures used by the 23 service providers. Small-scale service providers, those completing fewer than one job per working day, showed some similarities: a high proportion of pit latrine emptying (75% or above); a high proportion of manual jobs (75% or above); a high proportion of household customers (75% or above); manual emptiers paid per barrel emptied rather than a salary; renting rather than owning vehicles; not having an office; and not having permanent management (nonemptying) staff. Above this scale, service providers had more variety; two service providers in Kampala (K/R and K/N) reported completing more than 1000 manual emptying jobs per year while renting rather than owning vehicles. And the largest scale service provider in Kampala (K/F) reported a high proportion of household manual pit latrine emptying customers, in contrast to other large scale service providers. Across all scales, service providers reported having different approaches to using brokers to refer jobs; some only complete jobs referred by brokers, some identify their own jobs, and some do a mixture of both. Three service providers reported not completing any manual emptying jobs (B/Z, K/V and B/Y) and are mechanical only. All unit costs and cost structures are fully detailed in Supporting Information.

**Table 1:**
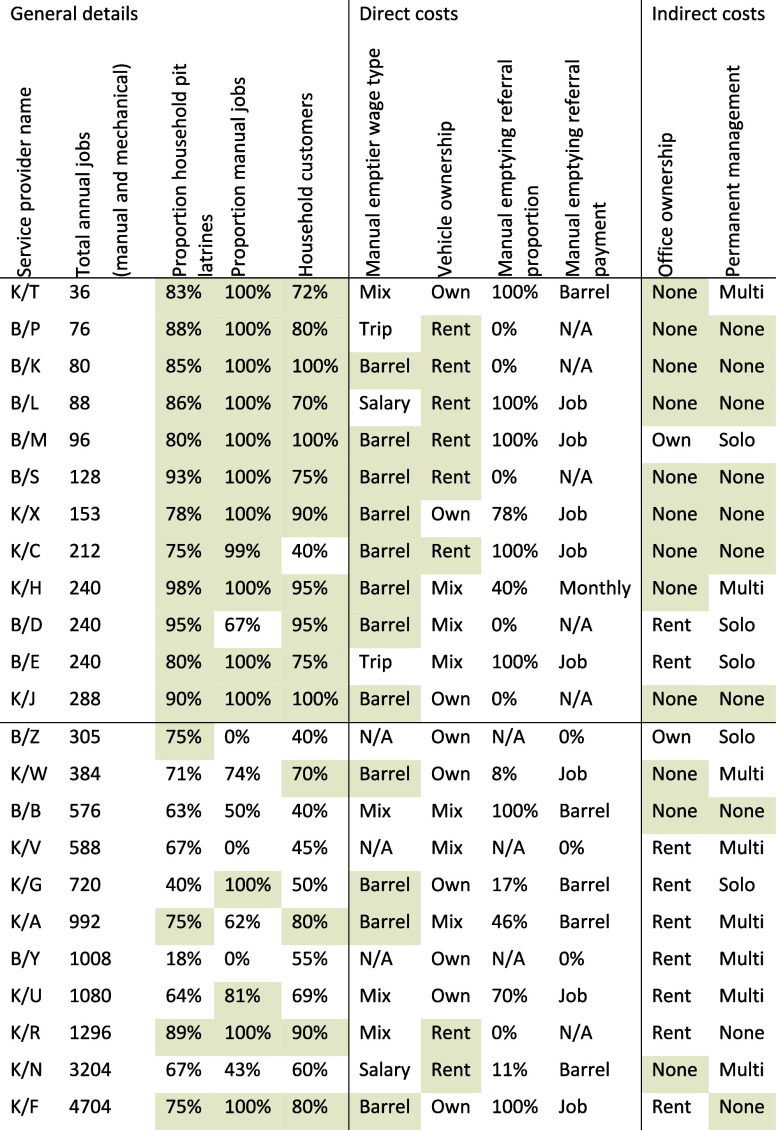
Faecal Sludge Emptying and Transport Service Provider Cost Structure Summary from Kampala, Uganda and Blantyre, Malawi, in Ascending Total Job Order^a^

a Characteristics associated with small-scale service providers (1 job per working day or lower) are shaded. Service providers are anonymized randomly with a city prefix: Kampala (K) and Blantyre (B).

Service providers reported different unit costs and cost structures for direct costs, including a mixture of owning and renting equipment and 13 service providers reported owning vehicles (ten own tricycles, seven own pickups and five own exhauster trucks). Most service providers paid manual emptiers per barrel (*n* = 16), with some (*n* = 2) paid per trip, or a monthly salary (*n* = 2). The reported average number of barrels emptied per job for each service provider varied between 78 in the high season and 2 barrels in the low season, and the median volume is 14 barrels in the high season and 6 barrels in the low season. Most service providers reported using 140 to 270 L barrels to transport sludge but three reported using 500 L barrels. Seven service providers reported renting pickups. Eight service providers reported fuel costs for pickups, with the median being 33 Int$_2020_ per trip.

All service providers reported at least some indirect costs. Fifteen service providers report using a broker to refer manual emptying customers, including seven where all manual jobs were referred by brokers. Brokers worked directly for a service provider or independently, and were paid either per barrel (*n* = 6), per customer (*n* = 8), or a monthly salary (*n* = 1). Sixteen service providers reported nonemptying staff salaries (permanent or temporary staff), with one service provider employing nine permanent nonemptying staff. The highest reported annual salary is almost 10,000 Int$_2020_ per year. Two service providers reported owning a building and nine reporting renting office space. The most common form of marketing was distributing business cards (*n* = 20), followed by other printed materials e.g., flyers (*n* = 14), with median total annual marketing expenditure 214 Int$_2020_. Only three out of 13 manual-only service providers reported any tax expenditure (corporation tax, value-added tax or withholding tax). In contrast, only two of the ten mechanical service providers did not report any tax expenditure. All service providers should have reported at least some tax expenditure. Overall, the median effective tax rate (as a proportion of total costs) among those reporting tax expenditure was 0.45% (*n* = 11). Service providers reported paying for various licenses: business, tax, city, public procurement and public assets authority, environment, water utility, and membership of the emptiers’ association. Ten service providers reported emptying water costs with no observable relationship to operating scale, and this is assumed to be a data artifact.

All manual emptying service providers in both cities reported paying dumping fees but these are excluded from analysis because they are a financial transfer between service providers and not the cost of emptying^25^ i.e., they are a cost incurred by service providers as part of their business model but they are not associated with the cost of emptying. In Kampala all manual emptying service providers transported sludge to a dumping bay next to treatment plant;^10^ previously a portion of sludge had been transported to mobile transfer stations but service providers preferred to use their own transport.^12^

### Manual Emptying Is Higher Annualized Cost than Mechanical Emptying When Operating at Full Capacity

Figure 1 compares the TACH breakdown for three emptying and transport methods operated at full capacity for the method (Scenario A) and at the same scale (Scenario B). Scenario A shows that manual emptying, using either a tricycle or pickup to transport sludge, is higher TACH than mechanical emptying using an exhauster truck when operated at full capacity. In contrast, Scenario B shows that when completing one job per day mechanical emptying is a similar TACH to manual emptying as the investment in more expensive vehicles and other fixed costs are distributed between fewer emptying jobs. This suggests that for small-scale service providers operating at about one job per day or fewer, using a tricycle is a cost-effective sludge transport option, despite requiring more trips to transport the same sludge volume to the disposal site as tricycles’ have a lower capacity (five barrels) compared to pickups (14 barrels).

**Figure 1:**
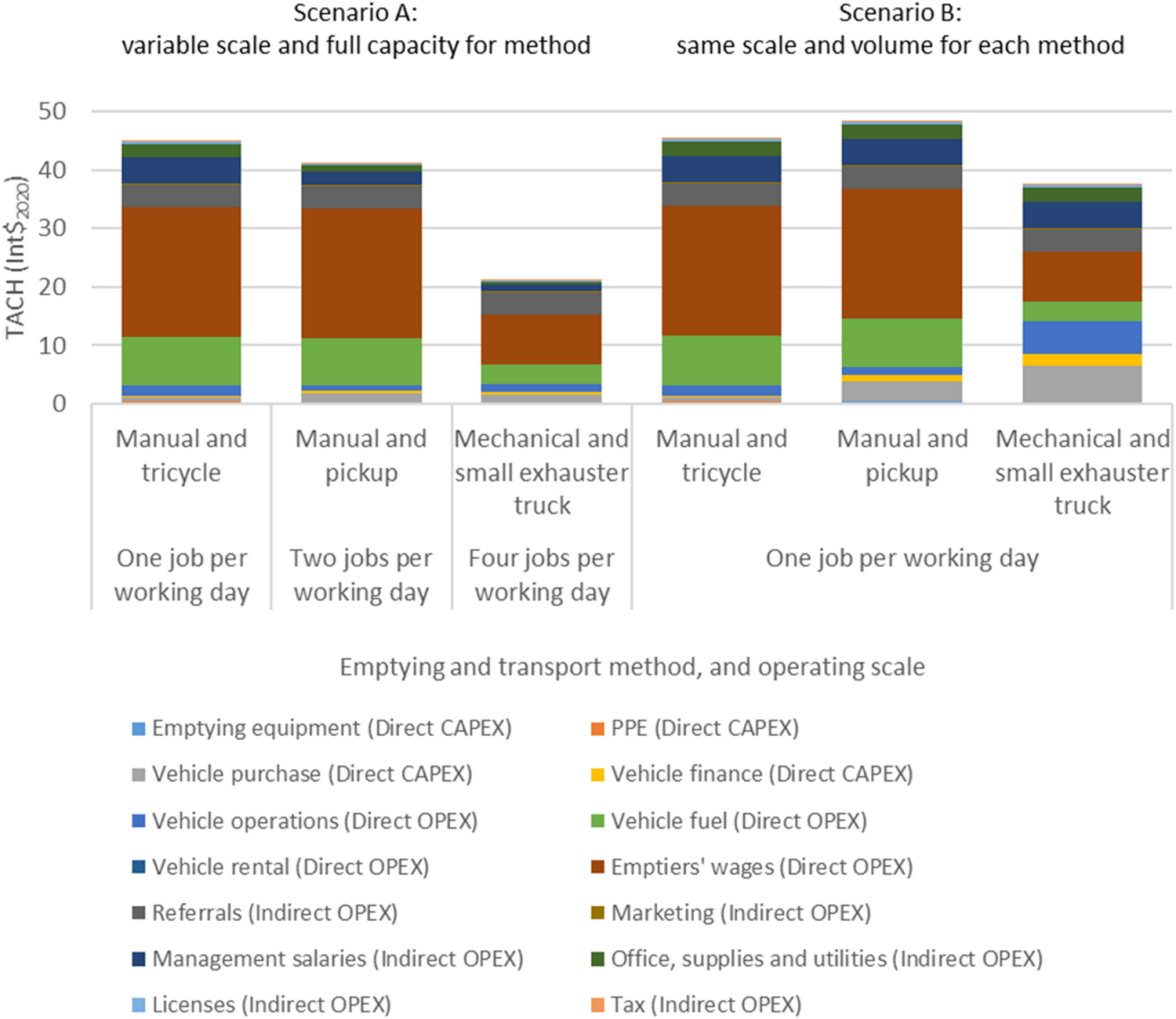
Total Annualized Cost per Household (TACH) breakdown for three faecal sludge emptying and transport methods operated at their respective full capacity (Scenario A) and the same scale (Scenario B: one job per day). Respective full capacities: manual emptying with a tricycle completing one job per working day; manual emptying with a pickup completing two jobs per working day; and mechanical emptying with an exhauster truck completing three jobs per working day. Based on a typical emptying job of 2.2 m^3^ (11 barrels) of sludge with a tricycle completing two trips to the treatment or disposal site per job, a pickup completing one trip to the disposal site per job, and a small exhauster truck (5 m^3^) having capacity for two jobs per trip to the disposal site. Costs are expressed in 2020 international dollars (Int$_2020_) based on annualized capital expenditure (CAPEX) and operational expenditure (OPEX).

Figure 1 also highlights the differences in cost structure between manual and mechanical emptying. For manual emptying, emptiers’ wages accounted for over half of TACH (22.30 Int$_2020_), in contrast to mechanical emptying (8.54 Int$_2020_), and the majority of the cost difference between the methods. For manual emptying with a pickup, fuel was the second largest individual cost voice after emptiers’ wages, transport overall was the second largest cost category (27%), and transport financing costs were only 1.3% of TACH. Indirect costs represented 19% of TACH.

CAPEX accounted for a small proportion of TACH at full capacity utilization for each method (5% for manual emptying using a pickup and 9% for mechanical emptying using an exhauster truck) which contrasts with other forms of water and sanitation infrastructure that are widely accepted to be higher CAPEX. As CAPEX was low for each method, variations in the discount rate would have a limited impact on TACH.

Additional costs related to formalization were 45% of TACH (personal protective equipment, transport, referrals, marketing, management salaries, office, licenses and tax). Higher costs for formal service providers increases prices, increasing demand for informal emptying which is a near a substitute service for households,^7,10,13^ effectively limiting uptake of formal services.

Manual emptying being higher TACH than mechanical emptying when operating at full capacity can be explained by manual emptying being more laborious and time-consuming.^6,14^ This is consistent with previous studies that found emptiers’ wages to be the largest cost category and manual emptying to be higher cost at the same scale compared to mechanical emptying.^13,23,25,32^ This is despite the higher capital investment in exhauster trucks, compared to tricycles and pickup trucks, because mechanical emptying is faster and can complete more jobs per day.

Emptiers’ wages representing over 50% of TACH for manual emptying represents an obvious cost minimization opportunity for service providers. Pit latrines are difficult to empty^5^ and there have long been suggestions to mechanize emptying,^3^ to improve both safety and efficiency. Reducing emptying times and increasing the number of jobs per day would increase vehicle utilization and reduce annualized costs. Some service providers reported using the Gulper and portable motorized technologies are emerging^7^ but uptake has been limited^11,12,33^ as emptiers find manual emptying more straightforward and it is often still required.^6^ Service providers in this study reported different approaches to employing manual emptiers by varying the number of emptiers, wages, and payment type (per job, per barrel or salary), and the interquartile range for total emptier wage was 6–16 $Int$_2020_ per 200 L barrel. In Rwanda, a service provider reported paying emptiers a monthly salary, which reduced TACH with increasing scale.^23^ While paying emptiers a salary rather than a wage may reduce cost at larger operating scales, paying emptiers per barrel may incentivise fast emptying.

Importantly, as a marginalized group in society there are also obvious concerns with reducing wages among sanitation workers,^34^ and there is a clear ethical tension between fairly compensating workers and minimizing costs.

All cost voices, emptying interval and emptying volumes are modeled independently for practical simplicity but some are likely to interact with each other. For example, comparison between three different methods is based on the maximum daily number of jobs for that combination of emptying method and transport. Manual emptying with a pickup rather than a tricycle can complete more jobs per day because larger sludge volumes can be transported per trip, fewer trips are required per job, and more jobs can completed in 1 day, irrespective of the emptying method. Similar, reducing the number of emptiers would likely reduce the maximum number of jobs per day.

Manual emptying being higher TACH than mechanical emptying is a vertical equity consideration because manual emptying is typically associated with informal settlements.^5,14^ With service providers transferring costs of emptying to households through user-fees,^10^ households with systems requiring manual emptying are contributing more financially to safely managed sanitation. City authorities could improve vertical equity by supporting households to upgrade latrines to be more easily emptied^9^ or by regulating prices. For example, Kampala Capital City Authority has piloted a subsidy to support households to upgrade latrines where the city authority fund substructure improvements to enable more cost-efficient emptying, and households fund superstructure improvements.^35^ In Rwanda, a social enterprise have implemented a cross-subsidy between different customer groups^19^ and in Kampala itself, some service providers have used profitable contracts with institutional customers to financially attenuate unprofitable and seasonal household emptying requests.^10^

### Annualized Costs Vary between Households Producing Inequitable Financial Contributions Toward Citywide Safely Managed Sanitation

Figure 2 shows the results from the modeling scenario on household emptying characteristics and the corresponding variation in TACH. Figure 2a shows that 80% of households had a TACH between 10 and 120 Int$_2020_. Five households had TACH above 200 Int$_2020_—these are the private systems emptying at the lowest emptying interval. Figure 2b shows the positive relationship between TACH and household emptying rates (*r* = 0.89). Figure 2c shows that households empty between two and 40 barrels per job, with households in Blantyre emptying larger volumes per job (nine barrels per job) than in Kampala (five barrels per job). Figure 2c also shows that emptying volumes increased with emptying intervals but are not directly proportional. Figure 2d shows that volumetric cost (the gradient in Figure 2b) varied between 50 and 250 Int$_2020_ per m3 and is inversely proportional to emptying volume per job: higher volume jobs reduce the volumetric cost.

**Figure 2:**
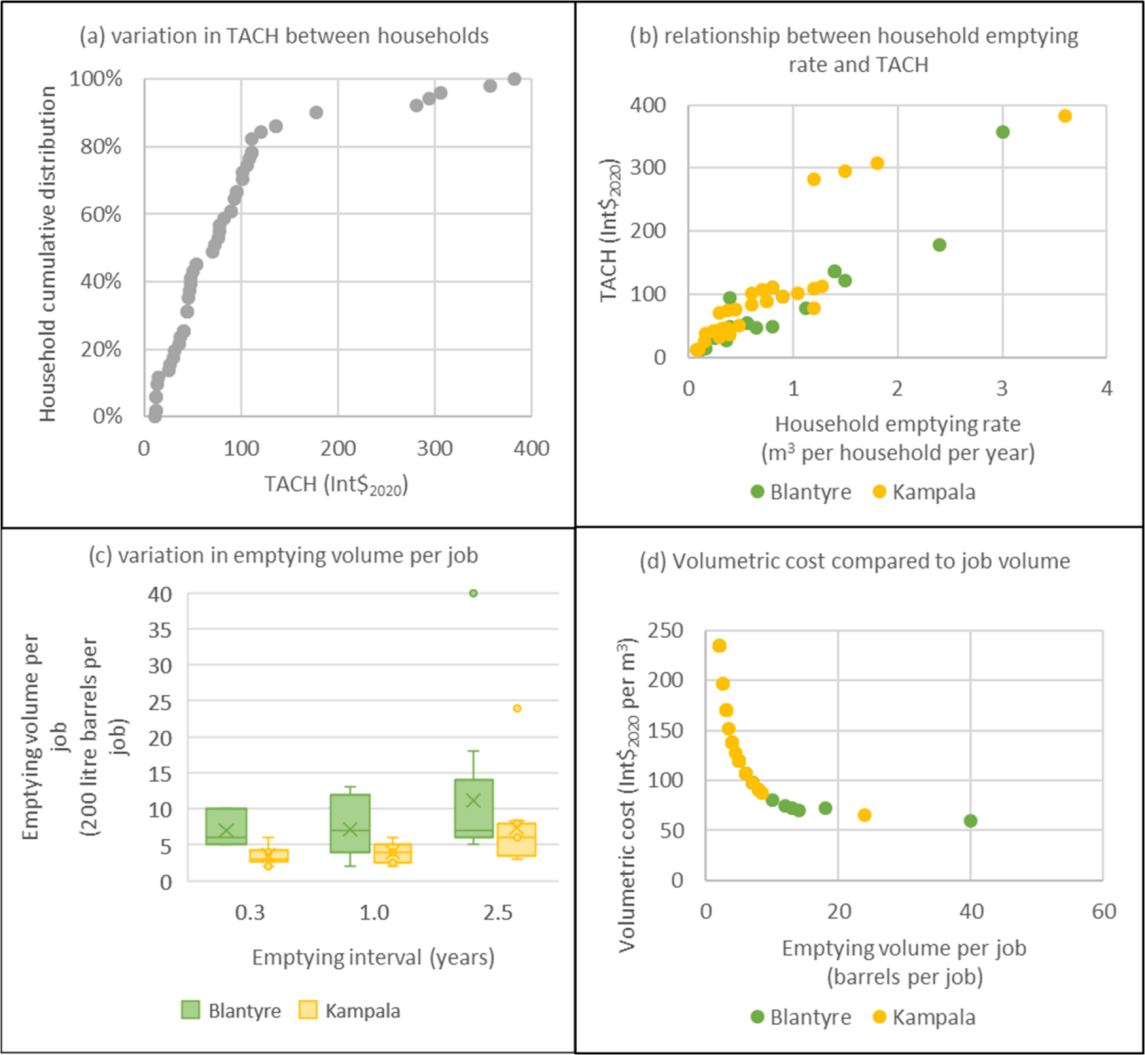
Modeling scenario 1: individual household (*n* = 52) emptying characteristics and Total Annualized Cost per Household (TACH) based on a manual emptying service provider completing two jobs per working day consisting of a single team using a pickup to transport sludge to treatment. TACH in expressed in international dollars (Int$_2020_).

Overall, this modeling scenario shows that the wide variation in TACH is largely related to both the annualized household emptying rate (Figure 2b) and emptying volume per job (Figure 2d). This is intuitive as the two largest cost voices for manual emptying (emptiers’ wages and vehicle fuel) are both directly related to emptying volumes. As service providers transfer the cost onto households through user-fees^7,10,17^ households are making horizontally inequitable financial contributions toward citywide safely managed sanitation based on their annualized household emptying rate and job volume. It could be argued that households using more water and consequently having higher annualized emptying rates should make a larger financial contribution. But at the same time, households using unlined pits are likely to have lower emptying rates due to infiltration reducing sludge volumes,^36^ and will make a lower financial contribution toward safely managed sanitation, while potentially polluting groundwater.

Surveyed households qualitatively reported the emptying interval from four options and these were converted to numerical values to allow the calculation of TACH and household emptying rate. “Every few years” was converted to 2.5 years but if it had been converted to a higher value (e.g., 3 years) this would have reduced TACH for those households in this modeling scenario but also reduced TACH overall but increasing the average emptying interval across all households. As TACH is directly related to the emptying interval it would be much more accurate to estimate based on service provider operational records that detail emptying intervals for repeat customers.

This study was unable to model the TACH variation caused by households being located different distances from service providers’ offices, and the treatment plant or disposal site. Travel distances are beyond household control and it is widely reported that service providers vary user-fees based on travel distance, time and fuel consumption^7,10,17^ which is another clear example of horizontal inequitable financial contributions toward citywide safely managed sanitation.

City authorities could enable households to empty higher volumes per job by providing a financing mechanism to either households or service providers to attenuate payment for high-volume emptying.^9^ This could improve horizontal equity between households in financial contributions toward safely managed sanitation.

### Service Providers Would Considerably Reduce Costs through Capacity Utilization

Figure 3 shows the effect on TACH of capacity utilization and operating scale for a manual emptying service provider using a pickup truck to transport sludge. Increasing the operating scale and capacity utilization for a single team from 100 (a common scale in the service provider survey) to 600 jobs per year (the maximum for a single team) reduced TACH by 46%. Further increasing scale to three teams completing 1600 jobs per year only reduced TACH by a further 3%. As modeled, variable costs such as fuel, emptiers’ wages and referrals were unaffected by scale and accounted for 83% of TACH for a single team operating at full capacity (600 jobs per year). Vehicle costs (purchase, finance and operations) reduced with capacity utilization and scale, and were minimized before a second team and vehicle were required to manage the additional jobs (above 600 jobs per year). Fully utilizing a pickup truck accounted for 42% of TACH reduction between 100 and 600 jobs per year. Other fixed costs (marketing, management, office, licenses and tax) accounted for the remainder of the TACH reduction.

**Figure 3:**
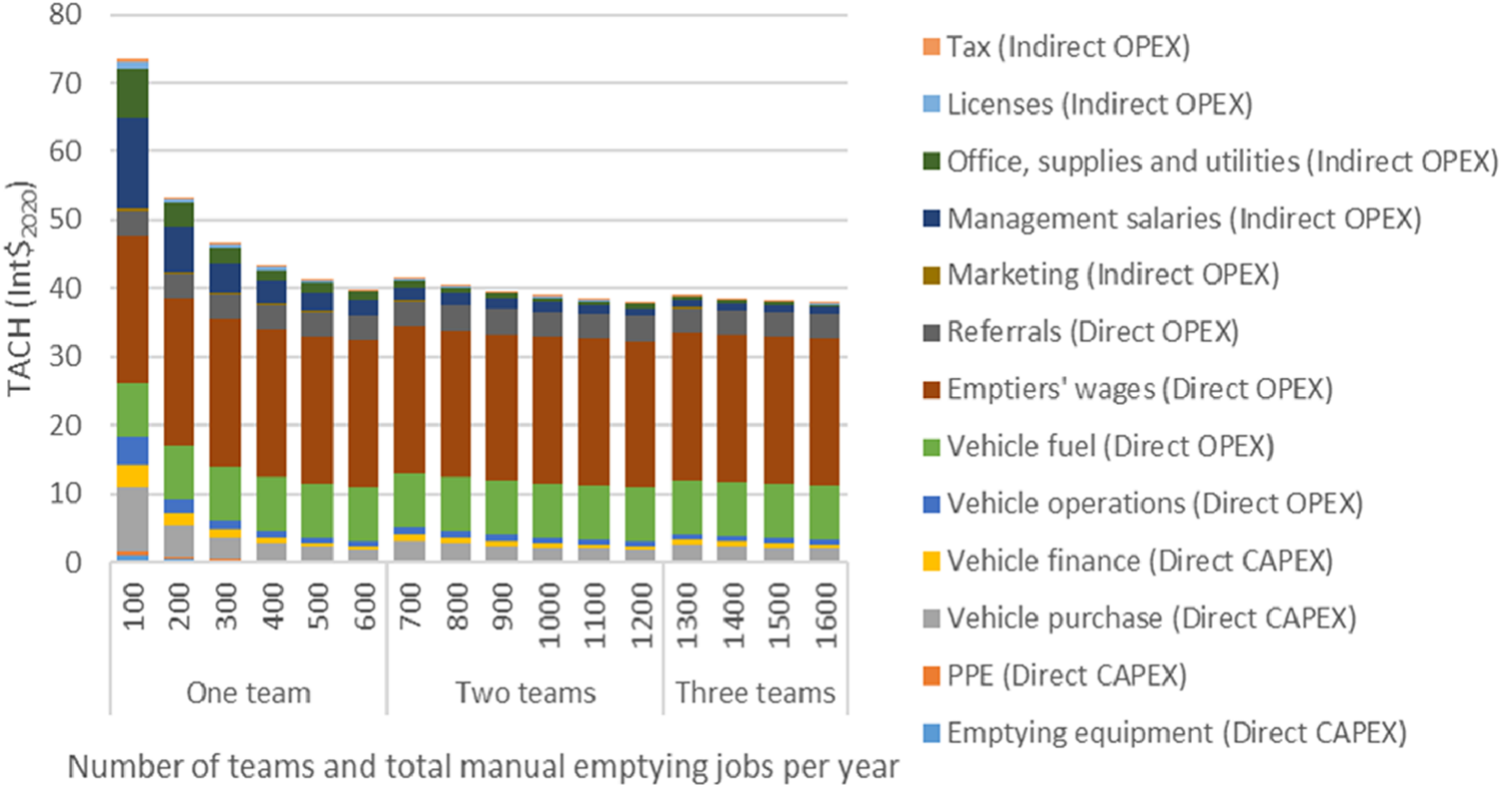
Modeling scenario 2: Total Annualized Cost per Household (TACH) and operating scale/capacity utilization of a manual emptying service provider using a pickup truck to transport sludge. Assumes an average 11 barrels per job with a maximum daily capacity of two jobs per team. Capital expenditure (CAPEX) and operational expenditure (OPEX) are expressed in international dollars (Int$_2020_).

Manual emptiers’ wages did not reduce with scale because they are modeled as being paid per barrel emptied but they would reduce with scale if paid as a salary—this is consistent with other studies.^23^ Similarly, fuel was modeled as a variable cost based on the number of trips to the disposal site, and because the number of trips remains constant with scale, the average fuel cost does not vary. However, in practice, service providers would be able to reduce average fuel costs while scaling by clustering emptying requests.^23,24^

Fully utilizing capacity to minimize costs is consistent with other analysis which found that operating sanitation systems at their design capacity is required to minimize TACH^25^ and for service providers to be profitable.^37^ Only three service providers (all in Kampala) report averaging more than 1.5 manual emptying jobs per vehicle per working day, highlighting that few service providers are fully utilizing their vehicle capacity. This low level of capacity utilization suggests that the market is both saturated and fragmented^10^ as service providers have significant spare capacity at current market prices, and that an open market structure is not cost minimizing for service providers.

Minimising TACH theoretically requires service providers to fully utilize equipment but practically they would also require capacity headroom to manage both seasonal variation and on-demand requests. Various suggestions have been made for service providers to minimize transportation costs, including: clustering on-demand requests;^23,24^ delegating emptying requests between service providers with spare capacity or more appropriate resources;^13^ transferring sludge between transportation vehicles to utilize spare capacity;^19^ permanent transfer stations;^38^ route optimization;^39^ or using higher volume barrel-based vehicles than tricycles and pickups such as flatbed trucks.^7,23^ These approaches may increase capacity utilization and efficiency but will introduce an indirect management cost^19^ not available in this data. Alternatively, proponents of scheduled desludging argue that workload predictability increases capacity utilization^40^ and provides revenue security which is linked to service efficiency.^13,32,41^

Owning rather than renting vehicles is often suggested by analysts as an option to reduce costs^42^ and is an aspiration of service providers.^10^ Renting a pickup had a 35% higher TACH than ownership when completing 600 jobs per year. The *financial tipping point* for owning a pickup was estimated to be 413 jobs per year and the *economic tipping point* was lower at 103 jobs per year because the financing costs were annualized over a longer time period (ten years) than the loan repayment (three years). Few service providers reported operating at a scale above the financial tipping point where it would be lower cost during the loan repayment period to finance pickup purchase through a loan. Financing vehicle ownership being higher cost than vehicle rental at the scales most manual emptying service providers operate presents a challenge because while renting vehicles is higher cost at larger scales, renting vehicles offers service providers flexibility to meet demand and has been linked to increased profitability.^10^

City authorities could recognize the challenges service providers face accessing loans^12,32^ and offer either lower price rental vehicles to small-scale service providers or support service providers to access better loan terms e.g., by acting as a guarantor. Similarly this has happened in the SWEEP project in Bangladesh where the city authority leased exhauster trucks to service providers on the contractual condition to serve low-income households.^43^

### Low Volume Emptying Can be Cost Minimized if Service Providers Cluster Emptying to Increase Capacity Utilization

Figure 4 shows two modeling scenarios on increasing emptying volume per job and proportionally reducing the emptying interval per job to reduce TACH by attenuating fixed costs. In both scenarios households were assumed to have a fixed annualized emptying rate (1.4 m^3^ per system per year) so that changes in emptying volume per job had a direct impact on the emptying interval and therefore cost annualization. In Scenario 3A the service provider was assumed to complete 300 jobs per year, irrespective of the emptying volume per job, and increasing the emptying volume minimizes TACH as the pickup transport capacity approaches full utilization. In this scenario emptying larger volumes had a lower TACH because fixed costs were attenuated over larger intervals. Higher volume emptying also increased capacity utilization and lowers some costs, which is consistent with others finding that partial emptying increases service provider costs by inefficiently using equipment^24,41^ and reduces profitability.^10^

**Figure 4:**
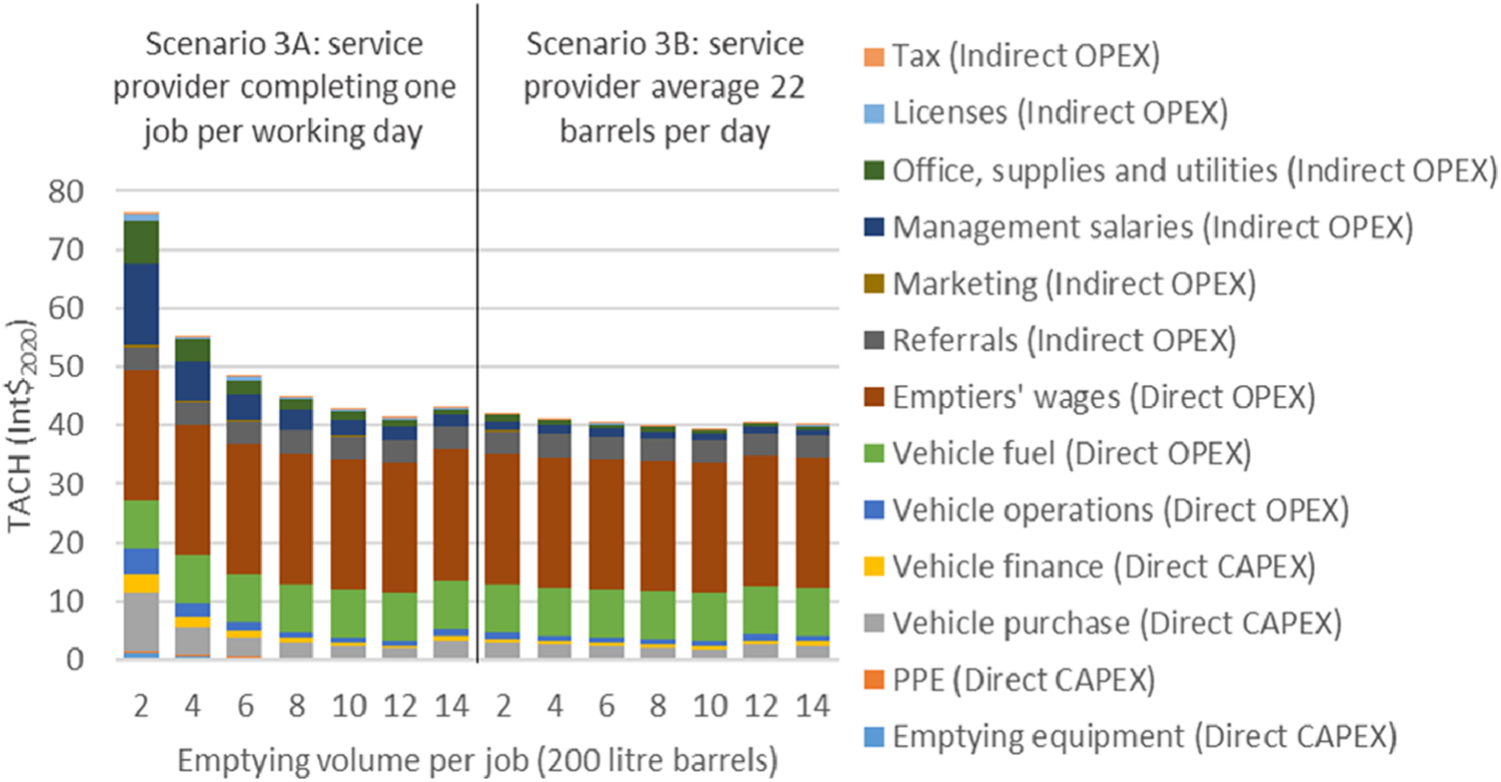
Modeling scenario 3: TACH and emptying volumes per job. Scenario 3A based on manual emptying service provider completing two jobs per working day with variable emptying volumes per day, scenario 3B based on a manual emptying service provider averaging 22 barrels per day with variable jobs per day. Annualized capital expenditure (CAPEX) and operational expenditure (OPEX) expressed in international dollars (Int$_2020_).

In Scenario 3B the service provider was assumed to empty an average of 22 barrels per day in total across all jobs i.e., Eleven jobs of two barrels or five jobs of 4.4 barrels. In Scenario 3B TACH was almost constant irrespective of the emptying volume per job because the service provider maintained a constant annual emptying volume with constant associated variable costs. In contrast to Scenario 3A, at lower emptying volumes, fixed costs were attenuated over a proportional number of additional households. In this scenario, emptying jobs were clustered (nearby households were grouped together) and additional travel costs were considered to be negligible. However, this does not account for the additional administrative costs of coordinating multiple emptying jobs.^19^

This modeling scenario suggests that if service providers are not fully utilizing their capacity, low-volume emptying would increase costs (Scenario 3A) but if service providers are able to efficiently coordinate emptying to fully utilize capacity, then costs could be minimized (Scenario 3B) with low-volume emptying jobs. As households prefer low-volume and low-price emptying to manage household budgets,^9^ and service providers have responded by using volumetric tariffs and minimum emptying volumes,^7,10,17^ there is therefore a tension between either high interval emptying to minimize TACH or low interval emptying to support household budgeting. Small scale service providers operating in competition are not incentivized to collectively coordinate and cluster emptying requests to minimize costs,^23,24^ and this reinforces the tension between prices and cost efficiency. All service providers surveyed reported using a per barrel tariff in response to demand for small volume, partial emptying.^7,10,17^ In both study-cities and elsewhere, the emptying interval is partly an outcome of market dynamics: households request emptying when pit latrines are full, service providers empty on-demand, and an affordable volume of sludge is removed.^7^

As in the other modeling scenarios, input variables were assumed to be independent but there is likely to be some relationship between emptying interval and emptying volumes because sludge reduces in volume while retained in pits. In other words, lowering the emptying interval may further increase the annualized cost as higher annualized sludge volumes would need to be emptied.

Cities have both demand and supply side options to manage this tension between low-price emptying and scaling efficiencies. Households could be supported to attenuate payment for higher interval emptying through a financing mechanism^9^ or through demand aggregation and semiplanned request clustering.^23,24^ Alternatively, service providers could be supported to achieve utilization efficiencies e.g., through scheduled emptying.^40^

### Limitations

The service providers are operating in a nascent system and are not representative of established, stable service providers. The service providers received business model support from the organization collecting the survey data which may have influenced their responses to encourage further support. The sample is not representative from either city and consists of the service providers that voluntarily participated in the survey. The sample size for some variables was very small and there are missing data e.g., where service providers did not report costs. Data was self-reported estimates which required less resources to produce compared to operational records but was likely subject to reporting bias. In combination these limitations reduce reliability^27^ and it is not known whether they have increased or decreased cost estimates. Analytical cost estimating introduces an optimizm bias and aggregates variable uncertainty.^44^

Similarly the household survey sampled users of the service providers supported by Water For People and were likely to be biased toward systems which can be more easily emptied by formal emptiers, and probably households with a higher ability to pay for formal emptying and consequently higher emptying volumes.^7^ In other words, the sample is likely to represent households that have a lower annualized cost because service providers can complete more jobs per day and empty larger volumes which increases capacity utilization.

Future research could be conducted on full-scale service providers operating in established systems and be based on their operational records to improve reliability e.g., the recent implementation of scheduled desludging in Wai and Sinnar, India.^40^ When the CACTUS database, an empirical cost database for urban sanitation systems that uses the TACH accounting metric, population increases it will be possible to perform parametric analysis across multiple service providers to better understand cost drivers,^8^ rather than using analytical estimates.

### Policy Implications

This study demonstrates that TACH varies considerably between households. As service providers transfer costs to households through user-fees, households are making variable financial contributions; this is both horizontally and vertically inequitable because safely managed sanitation is a public and merit good, and benefits are citywide.

Analysis showed that TACH variability is due to several factors. Manual emptying is higher TACH than mechanical emptying because it is more laborious and has higher wage costs, and because it is longer duration, fewer jobs can be completed per day and fixed costs are annualized across fewer jobs. Manual emptying being higher TACH than mechanical emptying is an example of vertical inequity as manual emptying is associated with informal settlements. Manual emptying service providers also report a fragmented market and lower operating scales with low levels of capacity utilization, which further increases TACH. At the same time, many service providers are operating at low scales where it is lower cost to rent vehicles than purchase using loans. Households have different annualized sludge emptying rates, causing large TACH variations. Households have a preference for low-volume emptying to minimize one-off payments but this would increase TACH if service providers do not coordinate emptying across multiple jobs to increase overall capacity utilization from low-volume emptying. Alternatively, high-volume emptying is lower TACH but would exclude households that cannot afford higher one-off payments. TACH variation between households using manual emptying services is an example of horizontal inequity as users of the same service are making different financial contributions. In both cities, households have a choice of emptying services but services are fragmented and service providers are not maximizing capacity utilization while managing seasonal demand fluctuations and competing with other service providers.

Service providers and city authorities have many options to incrementally increase coverage of formal manual emptying services using market-based service delivery mechanisms and to improve equity. Households could be supported to upgrade their sanitation systems to enable safe and cost-efficient emptying. Economic regulation could be introduced for price controls and/or a cross-subsidy to attenuate TACH differences between emptying methods and households. Zonal licensing or geographical contracts could aggregate emptying requests and promote capacity utilization. Scheduled desludging could provide certainty over future work while also promoting capacity utilization. Low-cost vehicle rentals to service providers could support them to manage demand surges. And a financing mechanism could support households to attenuate payment and incentivise cost-efficient and high-volume emptying. In addition, city authorities could use public funding to subsidize manual emptying to promote horizontal equity.

Alternatively, city authorities could take an alternative nonmarket-based, public service approach by determining the full citywide cost liability for citywide sanitation services; regulating to remove the direct user payment; frequently collecting user-fees and/or taxes rather than one-off payments e.g., a sanitation surcharge on water bills or a property tax to fund services; and either contracting services to the private sector or publicly delivering services. A public service approach would have multiple vertical and horizontal equity benefits of separating payment from the service type and quality, attenuating payment to help households manage budgets, enabling service providers to plan emptying and operate efficiently, establishing progressive rather than regressive user-fees, and distributing funding between variable cost services. Although adopting a public service approach for citywide services would be challenging because delivering services in informal settlements would be difficult to coordinate, particularly related to issues of land tenure and authorities having reliable data on households’ addresses. Further research and policy development would be required to determine equitable household financial contributions, while recognizing the differences in ability to pay, service quality, household size and emptying quantity, toward citywide safely managed sanitation.

This study demonstrates the cost variability between households for formal manual emptying services, and that this creates inequity between households, limiting progress toward citywide safely managed sanitation. Cities should consider the various regulatory options to increase coverage, including replacing direct user payments with an alternative and equitable funding model, based on the full citywide cost liability, to fund more efficient service delivery. Households with sewer connections do not pay every flush—why should households using pit latrines pay per empty?

## Supplementary Material



## Notes

Total annual manual emptying jobs and average emptying volume per job (Figure S1); hooks unit costs and number per manual emptying team (Figure S2); gulper unit costs and number per manual emptying team (Figure S3); barrel unit cost and number per manual emptying team (Figure S4); barrel volume (Figure S5); tricycle barrel capacity and unit cost (Figure S6); pickup barrel capacity and unit cost (Figure S7); manual emptier wages and team size (Figure S8); tricycle operational costs per vehicle (Figure S9); pickup operational costs per vehicle (Figure S10); pickup daily rental cost per vehicle (Figure S11); vehicle fuel costs (Figure S120); equipment costs per emptier (Figure S13); emptying water annual cost and total annual manual emptying jobs per year (Figure S14); broker referral costs and proportion of annual manual emptying customers referred by brokers (Figure S15); total annual marketing costs (Figure S16); total annual office consumables costs (Figure S17); total annual office rental costs (Figure S18); average management salaries (Figure S19); average license cost (Figure S20); total effective tax rate Service provider operational and financial properties (Figure S21); summary of service provider operational and financial properties Household manual emptying properties (Table S1); summary of household manual emptying statistics Modeling scenario on household emptying volume and interval (Table S2); cumulative distribution of household emptying volumes (Figure S22a); TACH compared to emptying interval (Figure S22b); TACH compared to annualized sludge emptying rate and emptying interval (Figure S22c); TAVC compared to emptying interval and city (Figure S22d); variation in TACH between cities and emptying intervals (Figure S22e); variation in household emptying rate (Figure S22f); TACH compared to annualized sludge emptying rate and city (Figure S22g); emptying volume per job compared to emptying interval and city (Figure S22h); cumulative distribution of household sludge emptying rate (Figure S22i); cumulative distribution of emptying volume per job (Figure S22j); TAVC compared to household emptying rate (Figure S22k); TAVC compared to job volume (Figure S22i) (PDF)
