# Supplementary material for: Formal Manual Fecal Sludge Emptying and Transport Services: Cost Analysis of 23 Service Providers in Malawi and Uganda
Source: Environ Sci Technol. 2026 May 20;60(21):15008–19. doi: 10.1021/acs.est.6c01385 (PMC13235543; doi:10.1021/acs.est.6c01385)
Supplement: Supplementary file 1 [file es6c01385_si_001.pdf]

Supporting information for: Formal manual faecal sludge emptying and transport services: cost analysis of 23 service providers in Malawi and Uganda

Author names

Jonathan D. T. Wilcox[1 2], Carlos E. V. Batarda[3], Julita C. Chinseu[4], Yvonne S. Lugali[3], Jamie K. Bartram[1], and Barbara E. Evans[1]*

Author addresses

1. University of Leeds, Leeds, LS2 9JT, United Kingdom
2. WaterAid, 6th Floor, 20 Canada Square, London, E14 5NN, United Kingdom
3. Water For People Uganda, Plot 15b Kitanta Close, Off Yusuf Lule Road, PO Box 4120, Kampala 25601, Uganda
4. Water For People Malawi, PO Box 1207, Blantyre, Malawi

* email: jonathanwilcox@wateraid.org

Data familiarisation

Service provider operating properties

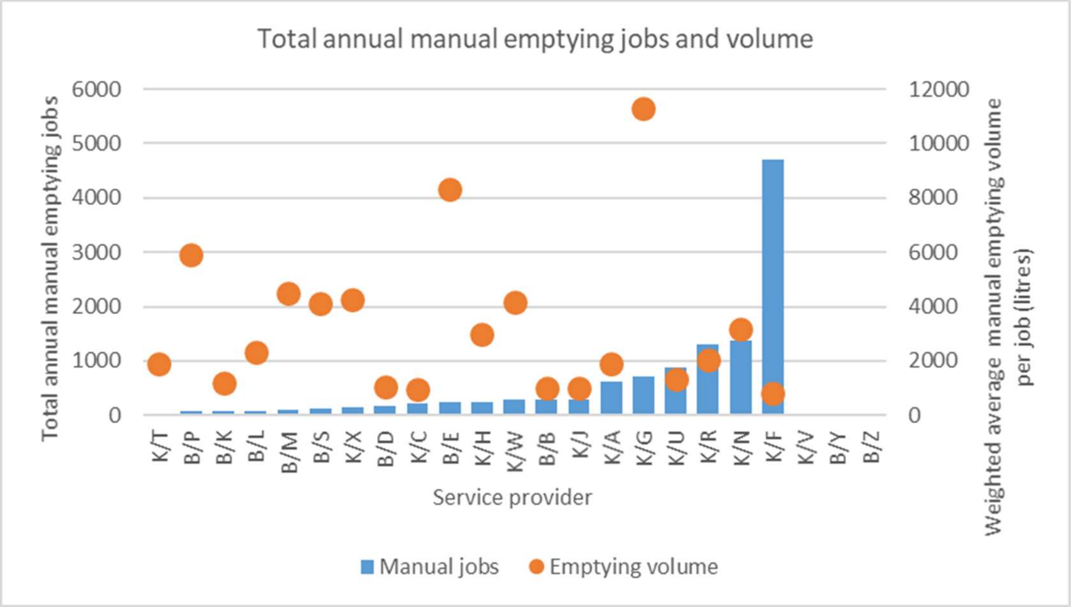

Figure S1 Total annual manual emptying jobs (bar on left vertical axis) and average emptying volume per job (dot on right vertical axis). Service providers ordered in ascending order of total annual manual emptying jobs. Service provider names are pseudonyms with a city prefix: Blantyre (B) and Kampala (K). Mechanical only emptying services providers are final three service providers.

Direct capital expenditure (Direct CAPEX)

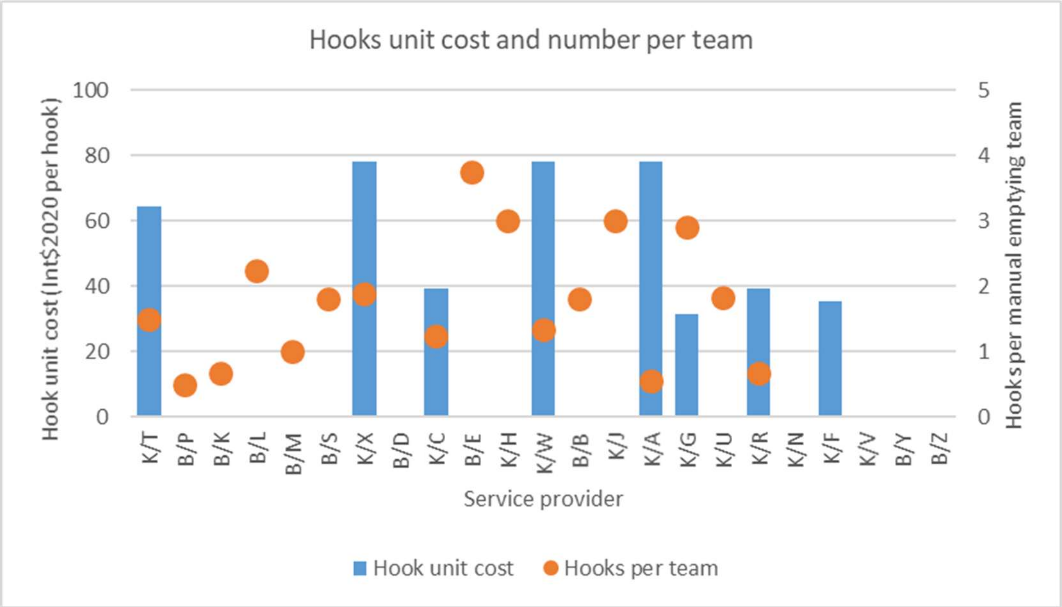

Figure S2 Hooks unit costs and number per manual emptying team. Service providers ordered in ascending order of total annual manual emptying jobs. Service provider names are pseudonyms with a city prefix: Blantyre (B) and Kampala (K). Mechanical only emptying services providers are final three service providers. Costs in 2020 international dollars (Int$2020).

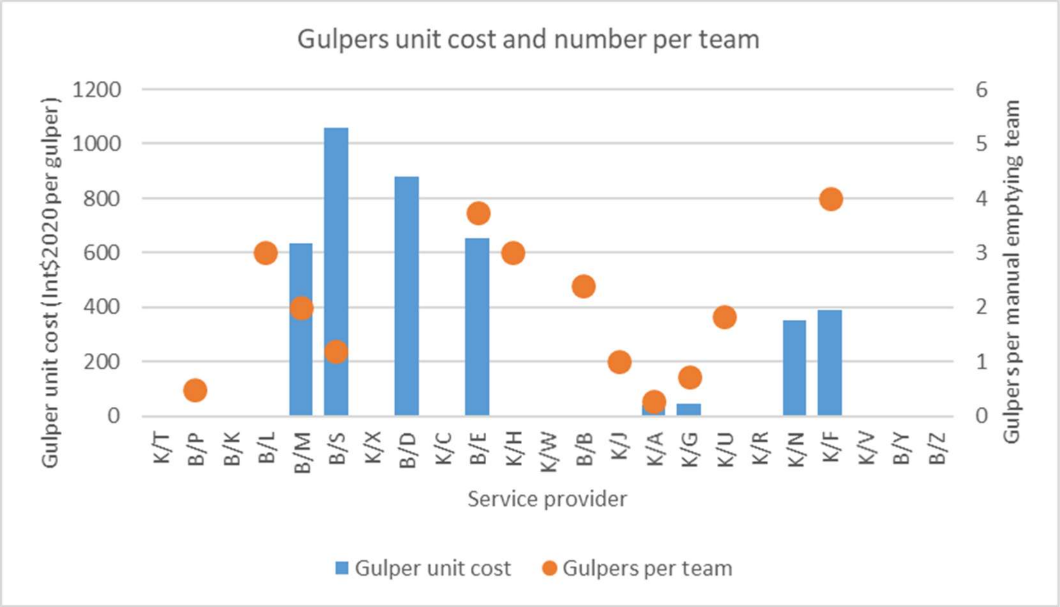

Figure S3 Gulper unit costs and number per manual emptying team. Service providers ordered in ascending order of total annual manual emptying jobs. Service provider names are pseudonyms with a city prefix: Blantyre (B) and Kampala (K). Mechanical only emptying services providers are final three service providers. Costs in 2020 international dollars (Int$2020).

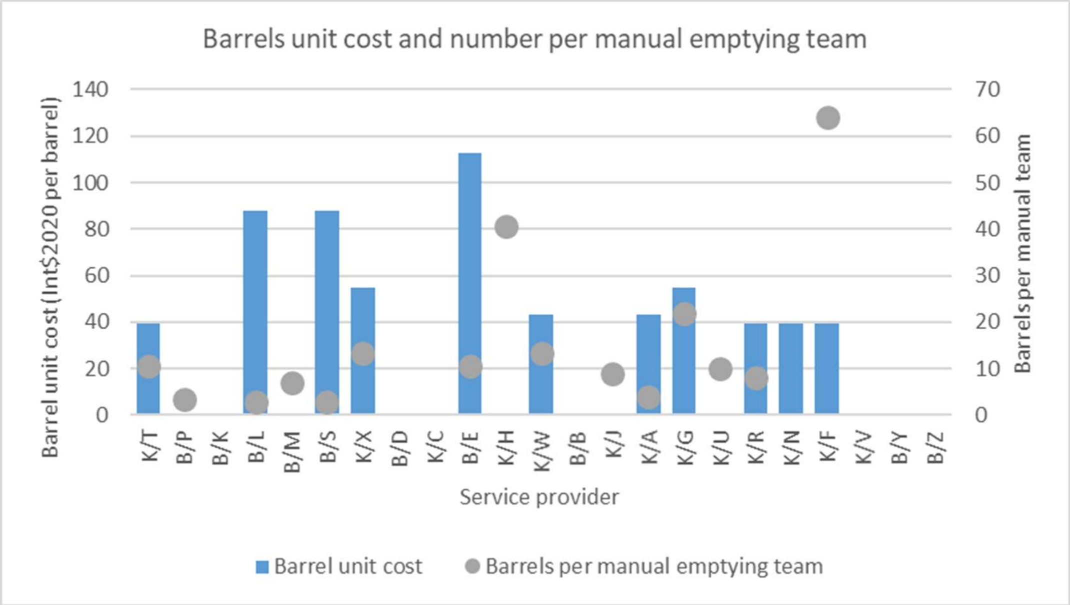

Figure S4 Barrel unit cost and number per manual emptying team. Service providers ordered in ascending order of total annual manual emptying jobs. Service provider names are pseudonyms with a city prefix: Blantyre (B) and Kampala (K). Mechanical only emptying services providers are final three service providers. Costs in 2020 international dollars (Int$2020).

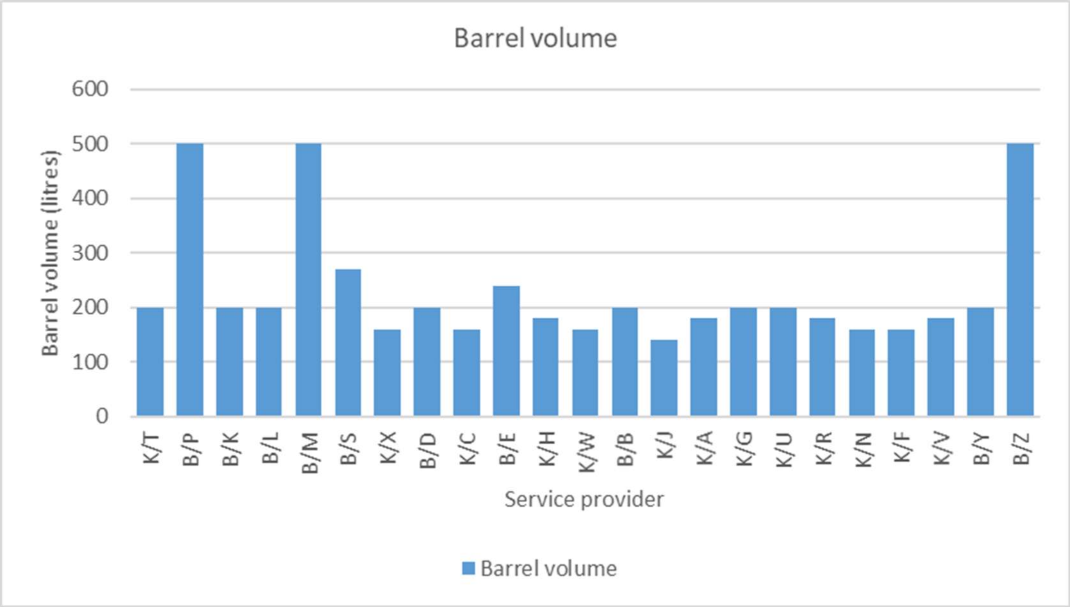

Figure S5 Barrel volume. Service providers ordered in ascending order of total annual manual emptying jobs. Service provider names are pseudonyms with a city prefix: Blantyre (B) and Kampala (K). Mechanical only emptying services providers are final three service providers. Costs in 2020 international dollars (Int$2020).

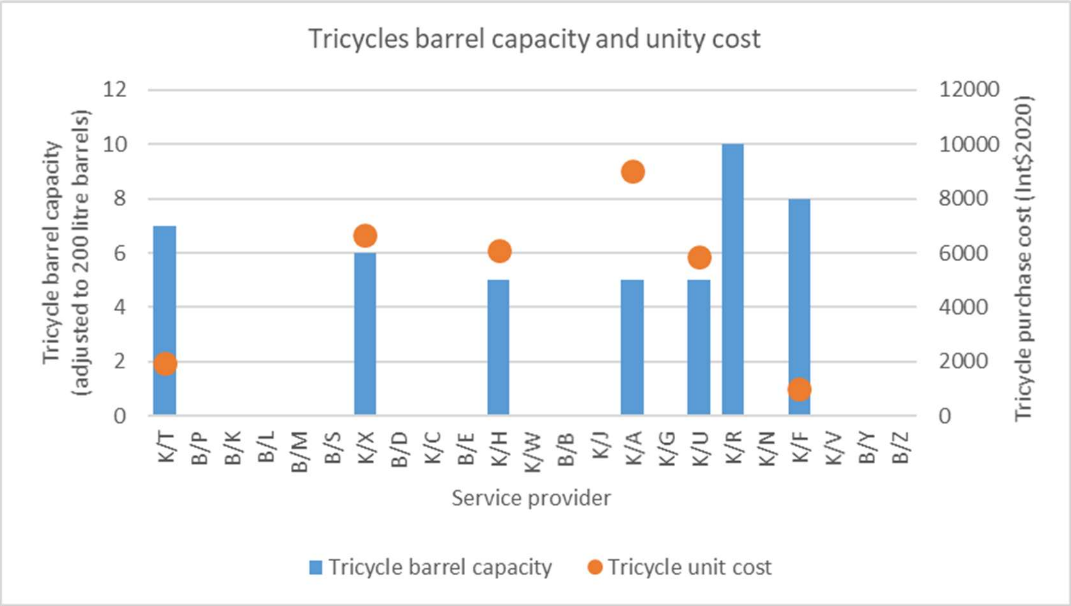

Figure S6 Tricycle barrel capacity and unit cost. Service providers ordered in ascending order of total annual manual emptying jobs. Service provider names are pseudonyms with a city prefix: Blantyre (B) and Kampala (K). Mechanical only emptying services providers are final three service providers. Costs in 2020 international dollars (Int$2020).

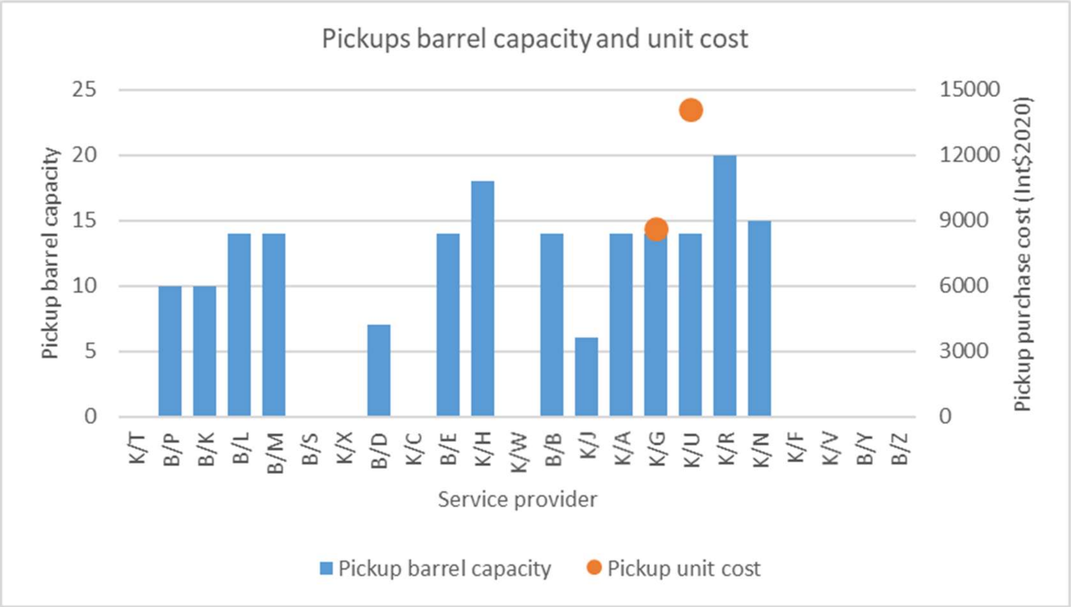

Figure S7 Pickup barrel capacity and unit cost. Service providers ordered in ascending order of total annual manual emptying jobs. Service provider names are pseudonyms with a city prefix: Blantyre (B) and Kampala (K). Mechanical only emptying services providers are final three service providers. Costs in 2020 international dollars (Int$2020).

Direct operational expenditure (Direct OPEX)

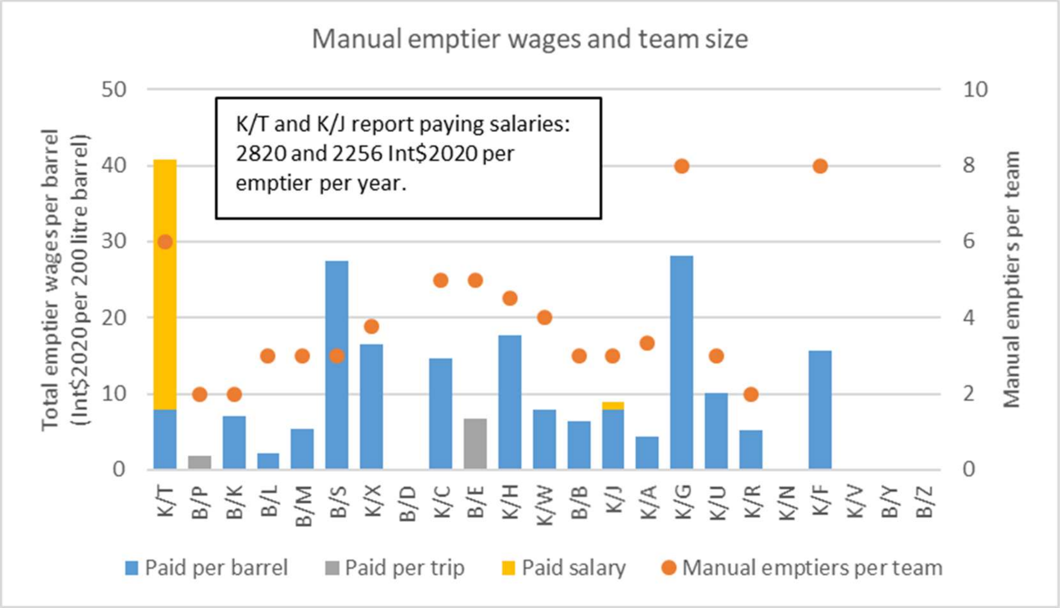

Figure S8 Manual emptier wages and team size. Emptier wages are normalised to per barrel and pro-rata based on a 200 litre barrel. Service providers ordered in ascending order of total annual manual emptying jobs. Service provider names are pseudonyms with a city prefix: Blantyre (B) and Kampala (K). Mechanical only emptying services providers are final three service providers. Costs in 2020 international dollars (Int$2020).

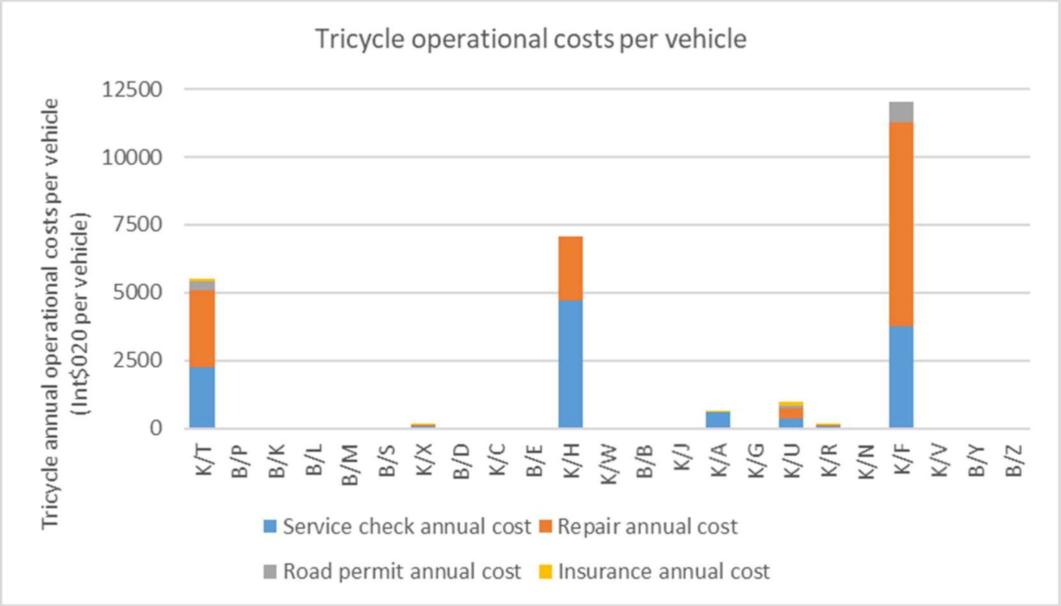

Figure S9 Tricycle operational costs per vehicle. Service providers ordered in ascending order of total annual manual emptying jobs. Service provider names are pseudonyms with a city prefix: Blantyre (B) and Kampala (K). Mechanical only emptying services providers are final three service providers. Costs in 2020 international dollars (Int$2020).

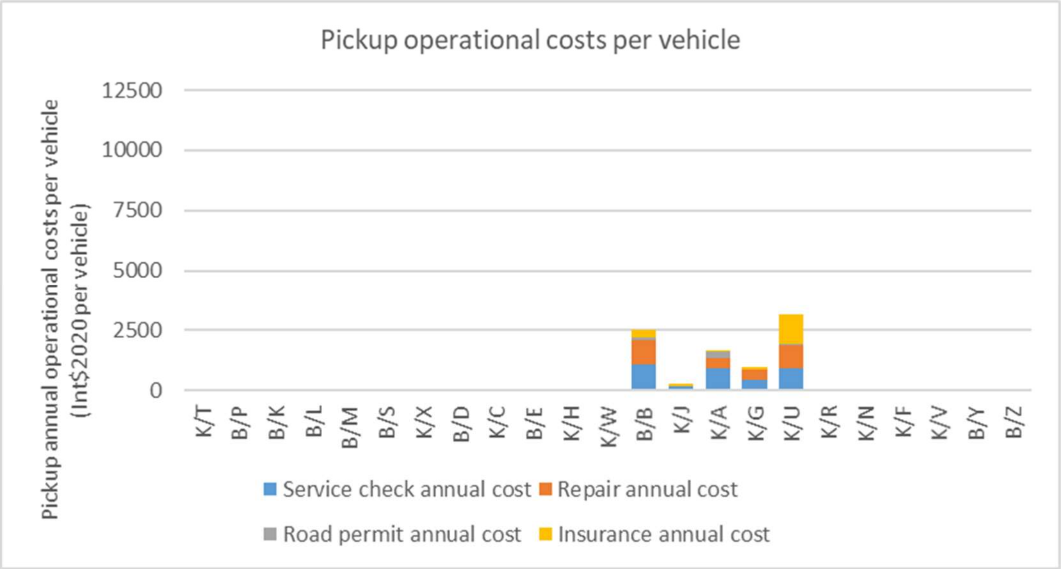

Figure S10 Pickup operational costs per vehicle. Service providers ordered in ascending order of total annual manual emptying jobs. Service provider names are pseudonyms with a city prefix: Blantyre (B) and Kampala (K). Mechanical only emptying services providers are final three service providers. Costs in 2020 international dollars (Int$2020).

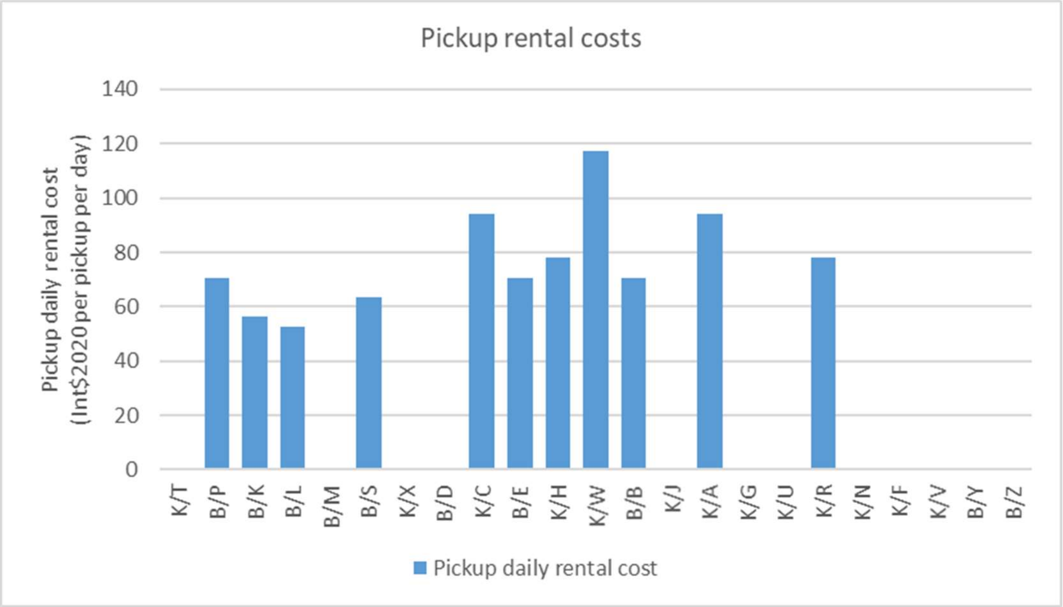

Figure S11 Pickup daily rental cost per vehicle. Service providers ordered in ascending order of total annual manual emptying jobs. Service provider names are pseudonyms with a city prefix: Blantyre (B) and Kampala (K). Mechanical only emptying services providers are final three service providers. Costs in 2020 international dollars (Int$2020).

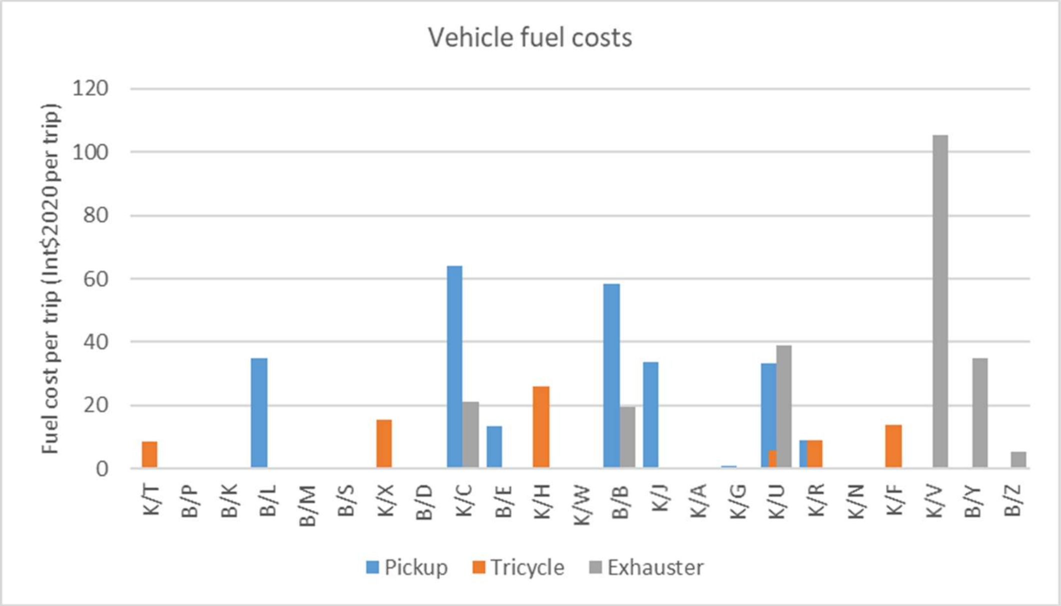

Figure S12 Vehicle fuel costs. Service providers ordered in ascending order of total annual manual emptying jobs. Service provider names are pseudonyms with a city prefix: Blantyre (B) and Kampala (K). Mechanical only emptying services providers are final three service providers. Costs in 2020 international dollars (Int$2020).

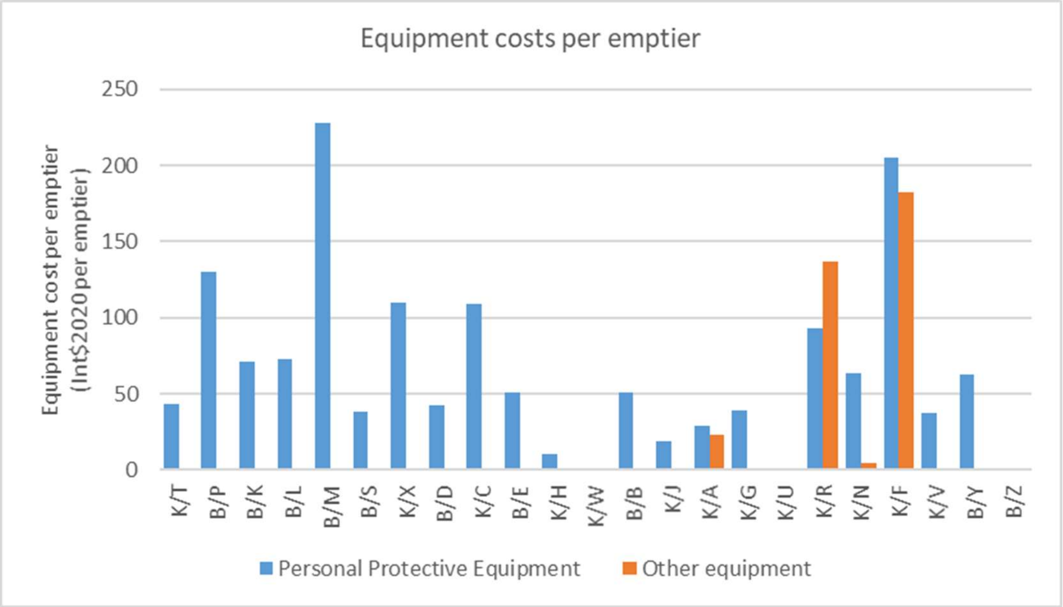

Figure S13 Equipment costs per emptier. Service providers are ordered in ascending order of total annual manual emptying jobs. Service provider names are pseudonyms with a city prefix: Blantyre (B) and Kampala (K). Mechanical only emptying services providers are final three service providers. Costs in 2020 international dollars (Int$2020).

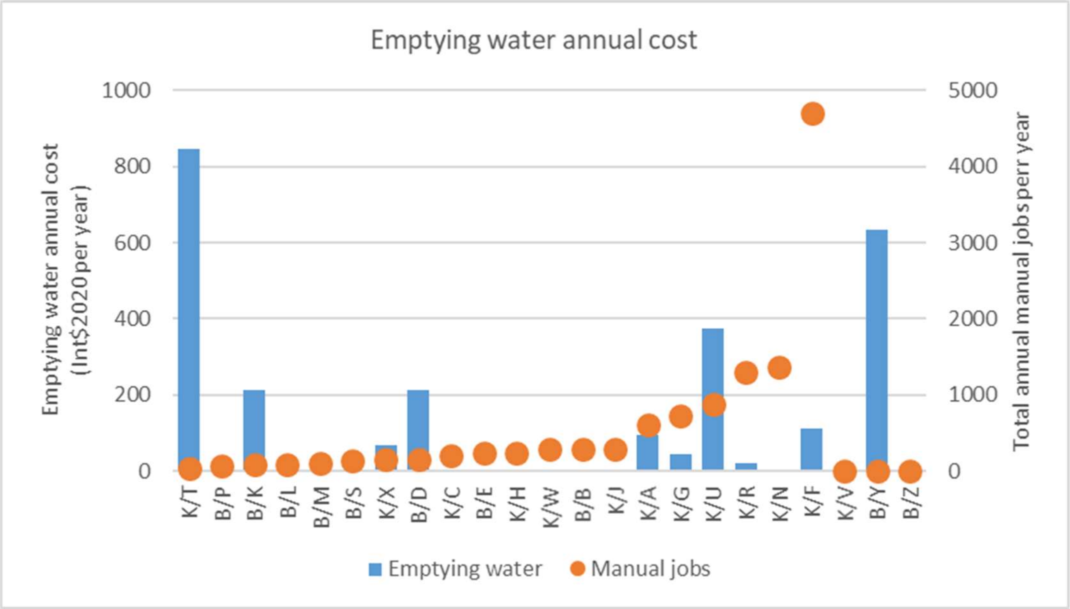

Figure S14 Emptying water annual cost and total annual manual emptying jobs per year. Service providers are ordered in ascending order of total annual manual emptying jobs. Service provider names are pseudonyms with a city prefix: Blantyre (B) and Kampala (K). Mechanical only emptying services providers are final three service providers. Costs in 2020 international dollars (Int$2020).

Indirect operational expenditure (Indirect OPEX)

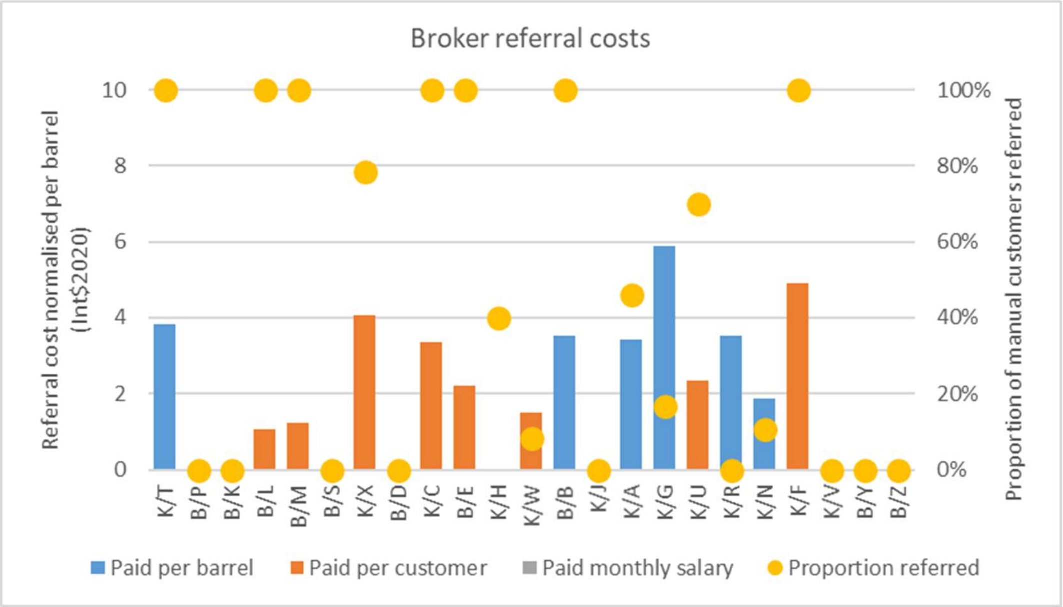

Figure S15 Broker referral costs and proportion of annual manual emptying customers referred by brokers. Service providers are ordered in ascending order of total annual manual emptying jobs. Service provider names are pseudonyms with a city prefix: Blantyre (B) and Kampala (K). Mechanical only emptying services providers are final three service providers. Costs in 2020 international dollars (Int$2020).

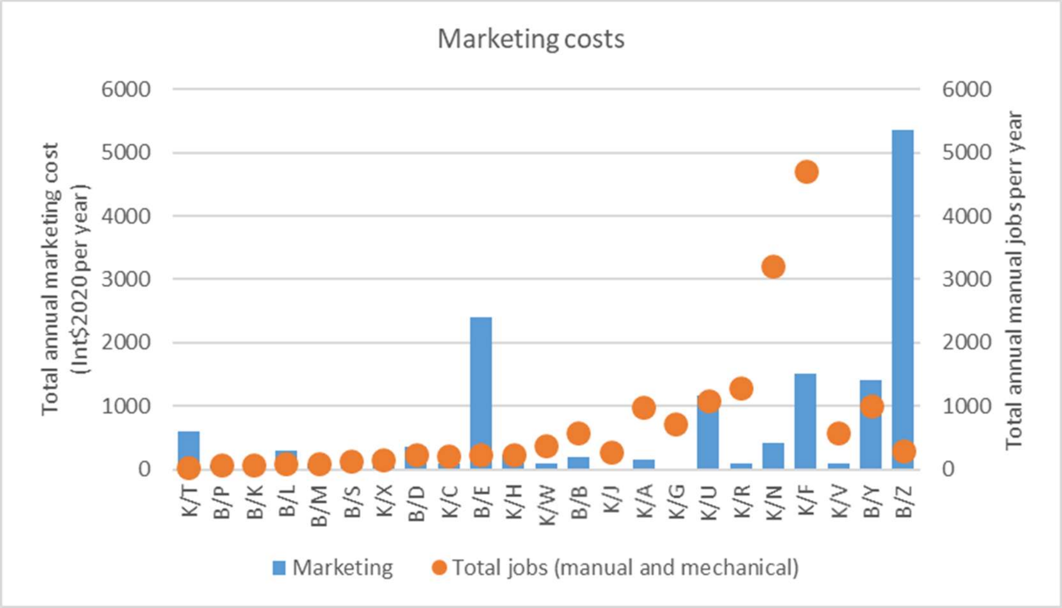

Figure S16 Total annual marketing costs. Service providers are ordered in ascending order of total annual manual emptying jobs. Service provider names are pseudonyms with a city prefix: Blantyre (B) and Kampala (K). Mechanical only emptying services providers are final three service providers. Costs in 2020 international dollars (Int$2020).

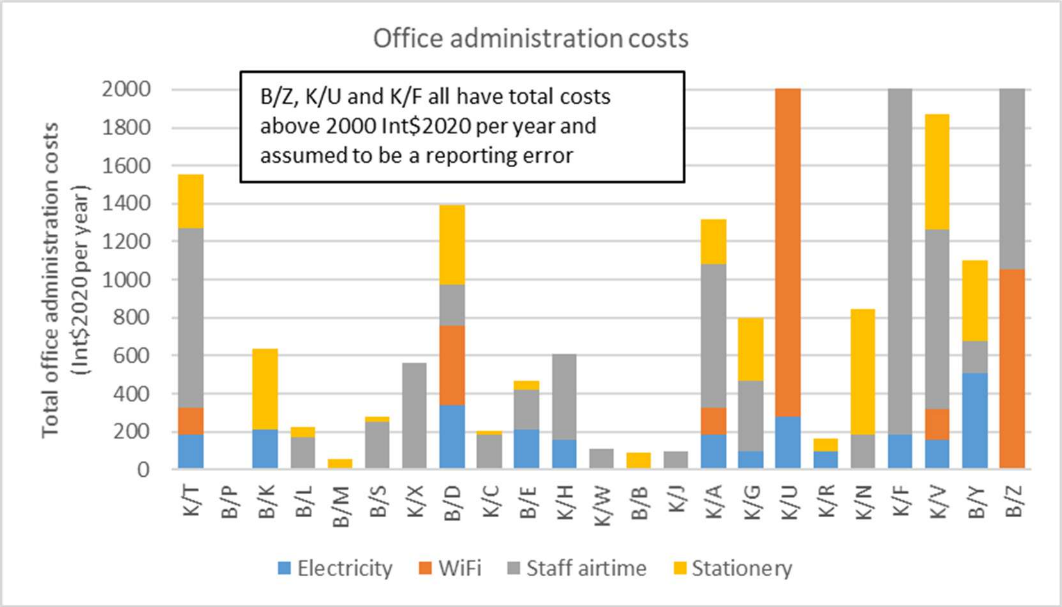

Figure S17 Total annual office consumables costs. Service providers are ordered in ascending order of total annual manual emptying jobs. Service provider names are pseudonyms with a city prefix: Blantyre (B) and Kampala (K). Mechanical only emptying services providers are final three service providers. Costs in 2020 international dollars (Int$2020).

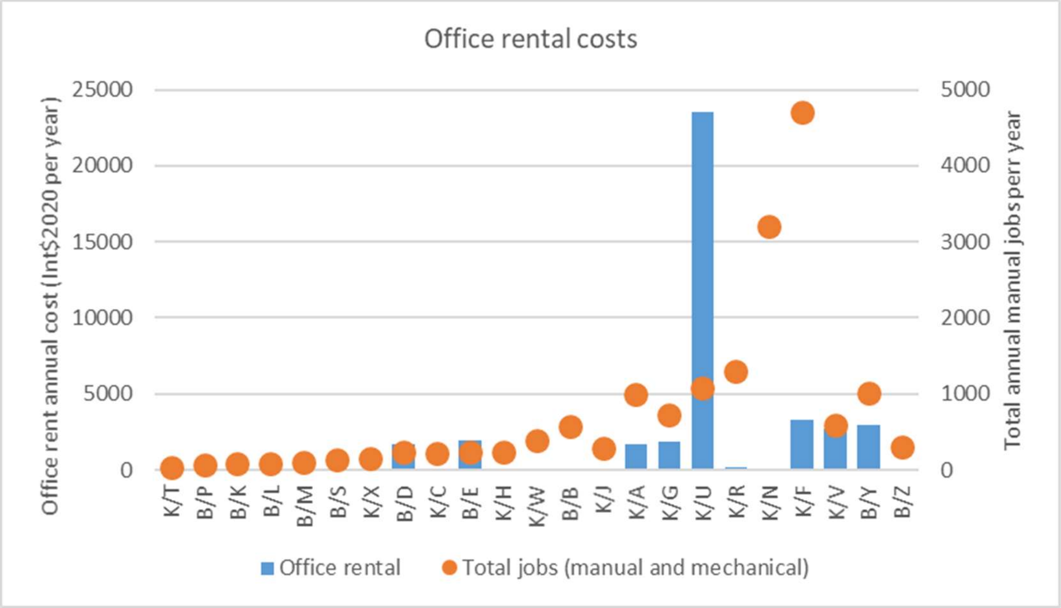

Figure S18 Total annual office rental costs. Service providers are ordered in ascending order of total annual manual emptying jobs. Service provider names are pseudonyms with a city prefix: Blantyre (B) and Kampala (K). Mechanical only emptying services providers are final three service providers. Costs in 2020 international dollars (Int$2020).

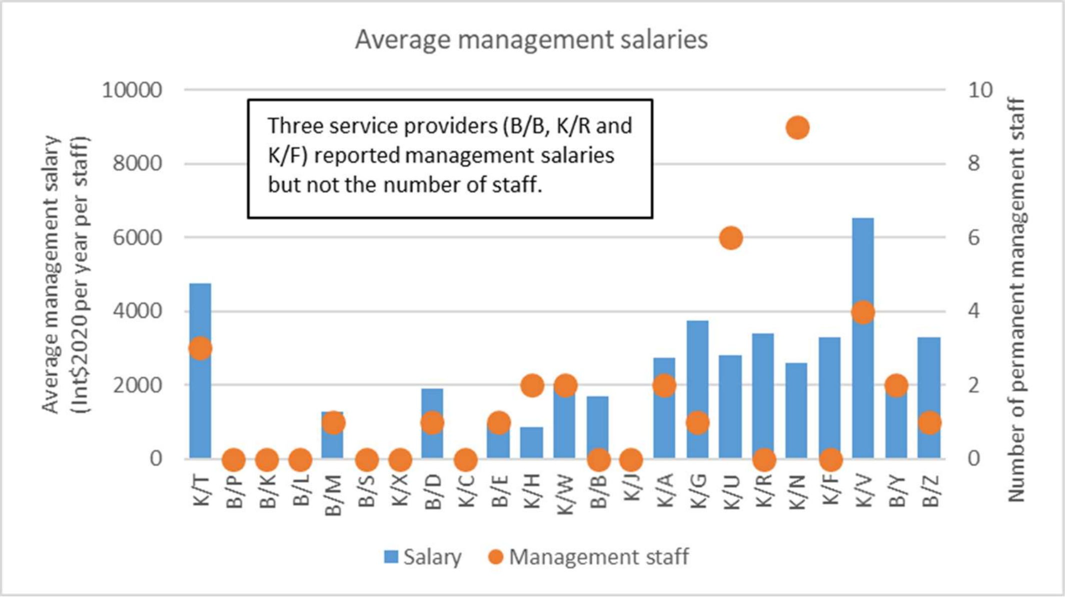

Figure S19 Average management salaries. Service providers are ordered in ascending order of total annual manual emptying jobs. Service provider names are pseudonyms with a city prefix: Blantyre (B) and Kampala (K). Mechanical only emptying services providers are final three service providers. Costs in 2020 international dollars (Int$2020).

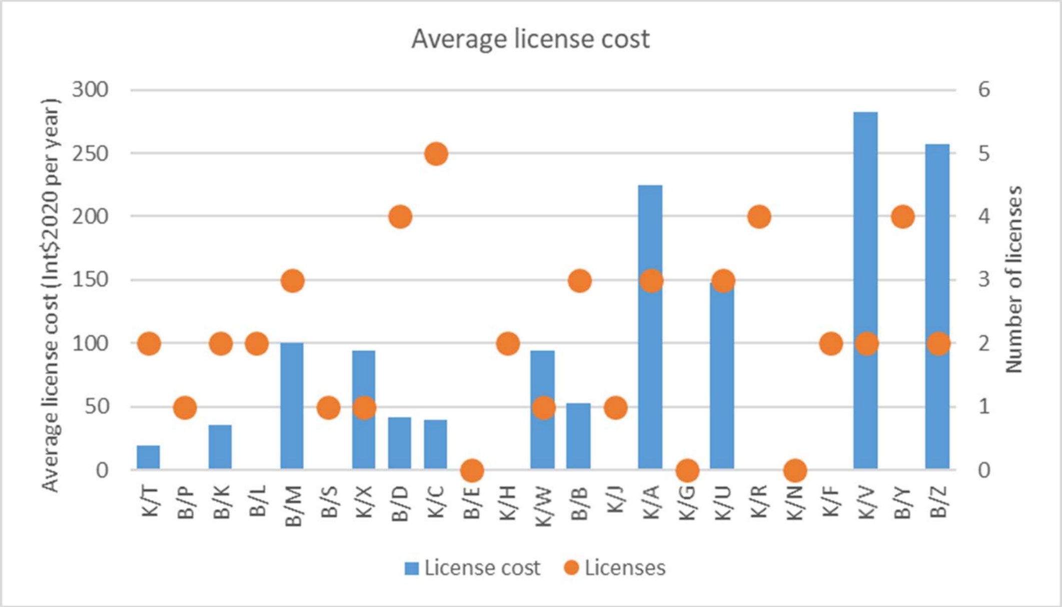

Figure S20 Average license cost. Service providers are ordered in ascending order of total annual manual emptying jobs. Service provider names are pseudonyms with a city prefix: Blantyre (B) and Kampala (K). Mechanical only emptying services providers are final three service providers. Costs in 2020 international dollars (Int$2020).

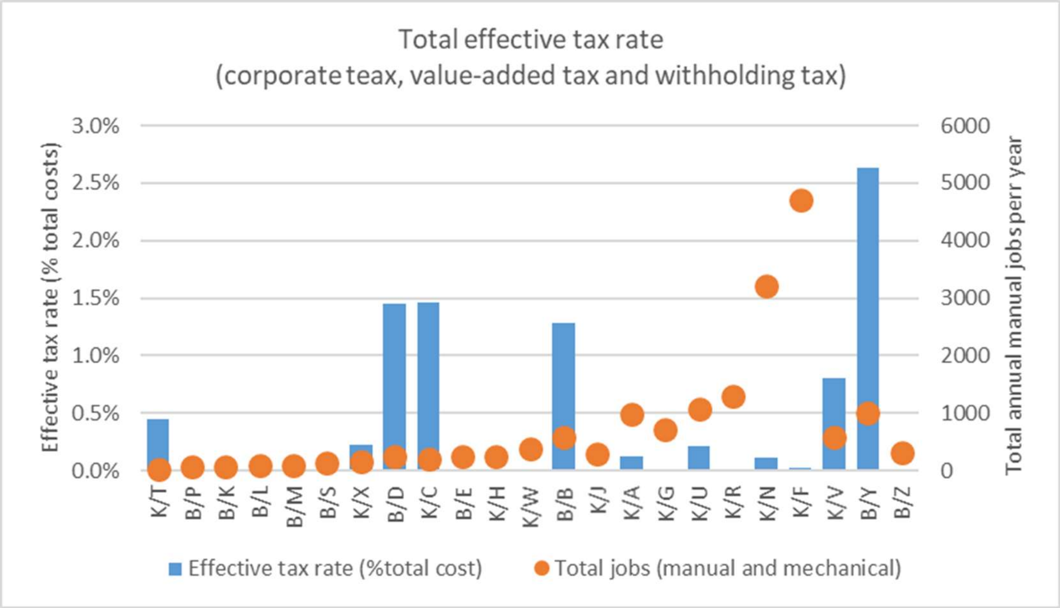

Figure S21 Total effective tax rate (all reported annual taxes as a proportion of total annual expenditure). Service providers are ordered in ascending order of total annual manual emptying jobs. Service provider names are pseudonyms with a city prefix: Blantyre (B) and Kampala (K). Mechanical only emptying services providers are final three service providers. Costs in 2020 international dollars (Int$2020).

Service provider operational and financial properties

Table S1 Summary of service provider operational and financial properties (operating properties, direct capital expenditure (CAPEX), direct operational expenditure (OPEX) and indirect OPEX) from Blantyre (BLZ) and Kampala (KLA). Units are shown in brackets. Costs are expressed in 2020 international dollars (Int$$_{2020}$). Sample size is the number of service providers reporting a value. Values are medians unless otherwise stated.

Category	Description	Value	Sample size	BLZ	KLA	25%ile	75%ile
Service providers operating properties							
Total annual manual jobs per service provider (manual jobs per year)		240	20	92	288	84	450
Mean emptying volume per job (litres per job)		2187	20	3226	1987	1165	4194
Mean number trips to disposal per manual job (trips per job)		1.1	20	0.8	1.2	0.7	1.3
Median proportion households customers (-)		75%	23	79%	80%	58%	90%
Direct CAPEX							
Physical assets	Hook unit cost (Int$$_{2020}$ per hook)	52	8	-	52	38	78
	Hooks per team (-)	1.1	21	1.0	1.4	1.1	1.9
	Gulper cost (Int$$_{2020}$ per Gulper)	513	8	766	200	276	709
	Gulpers per team (-)	1.3	15	1.6	1.3	0.9	2.1
	Barrel cost (Int$$_{2020}$ per barrel)	43	11	88	41	39	71
	Barrel volume (litres)	200	23	220	180	170	200
	Barrels per manual emptying team (-)	8.6	17	5.1	13.3	5.5	11.1
	Tricycle barrel capacity (number of 200 litre barrels)	5	7	-	5.0	4.7	6.7
	Tricycle purchase cost (Int$$_{2020}$)	5992	6	1958	6110	2937	6521
	Pickup barrel capacity (number of 200 litre barrels)	14	14	14	14	12.2	16.7
	Pickup purchase cost (Int$$_{2020}$)	31331	-	-	-	23499	39164

	Small (5 m^3) exhauster truck cost (Int$_{2020}$)	58746	-	-	-	39164	78329
Taxes and financing	Pickup total financing total cost (Int$_{2020}$)	10570	-	-	-	7928	13213
	Small exhauster truck total financing cost (Int$_{2020}$)	19819	-	-	-	13213	26426
Direct OPEX							
Salaries	Total emptier wages per 200 litre barrel (Int$_{2020}$ per 200 litre barrel)	8.4	18	6.3	14.7	6	16
	Annual salary per manual emptier (Int$_{2020}$ per year)	2538	2	-	2538	2397	2679
	Manual emptiers per team (-)	3.2	18	3.0	4.0	3.0	4.9
Equipment, Land and Buildings	Pickup annual service check cost (Int$_{2020}$ per year)	902	5	1057	667	431	940
	Pickup annual repair cost (Int$_{2020}$ per year)	705	4	1057	470	460	969
	Pickup annual road permit cost (Int$_{2020}$ per year)	88	3	88	137	64	162
	Pickup annual insurance cost (Int$_{2020}$ per year)	117	5	345	117	117	345
	Pickup rental per day (Int$_{2020}$ per day per vehicle)	34	7	34	40	21	65
	Other equipment costs per emptier (Int$_{2020}$ per year)	7.35	7	-	89.38	23	137
Consumables	Pickup fuel cost per trip (Int$_{2020}$ per trip)	33	8	35	33	12	41
	Tricycle fuel cost per trip (Int$_{2020}$ per trip)	11	6	N/A	11	9	15
	Exhauster truck fuel cost per trip (Int$_{2020}$ per trip)	28	6	19	39	20	38
	Personal Protective Equipment cost per emptier (Int$_{2020}$ per emptier)	57	20	63	43	39	97
	Emptying water (sludge fluidisation) annual cost (Int$_{2020}$ per year)	162	10	211	94	74	335
	Broker referral cost per barrel (Int$_{2020}$ per barrel referred)	3.3	15	1.7	3.4	1.7	3.7

Wages or commissions paid to staff on a variable or casual basis	Proportion of customers referred by brokers (-)	43%	20	50%	43%	41%	100%
Indirect OPEX							
Salaries	Management staff annual salary (Int$\$_{2020}$ per person per year)	2820	41	1797	2820	1691	3290
	Management staff per service provider (-)	2.0	13	1.0	2.5	1.00	3.00
Equipment, Land and Buildings	Office rent annual cost (Int$\$_{2020}$ per year)	1902	9	1902	2311	1692	2959
Consumables	Office electricity annual cost (Int$\$_{2020}$ per year)	188	12	275	172	157	229
	Office WiFi annual cost (Int$\$_{2020}$ per year)	294	6	740	153	147	898
	Staff airtime annual cost (Int$\$_{2020}$ per year)	315	18	211	508	188	893
	Office stationery annual cost (Int$\$_{2020}$ per year)	282	17	88	305	56	423
Services	Marketing annual cost (Int$\$_{2020}$ per year)	214	22	247	164	92	511
Administrative Fees, Taxes and Financing	Total tax as proportion of total costs (-)	0.5%	11	1.5%	0.2%	0.2%	1.4%
	Annual cost per license (Int$\$_{2020}$ per license per year)	94	15	53	94	41	167
	Maximum licenses per service provider (-)	5	20	4	5	1.8	3.0

Household manual emptying characteristics

Households across both cities reported different emptying characteristics: 78% of households reported having emptied their sanitation system (n=232); 88% of households using pit latrines reported emptying manually (n=144); 56% of households emptying manually reported sharing systems with other households (n=88); the mean emptying interval was 1.5 years (n=98) for pit latrines emptied manually; and the mean manual emptying volume was 1.2 m^3 per job (n=60) or the equivalent of six 200 litre barrels. About half of households empty volumes transportable in a single trip by a tricycle and the other half volumes transportable by pickup truck.

Table S2 Summary of household manual emptying statistics. Manual emptying interval quantitative conversion: every month = 0.08 years; few times per year = 0.33 years; once per year = 1 year; every few years = 2.5 years.

City	Combined			Blantyre			Kampala		
Emptying method	Manual	Mixed	Mech	Manual	Mixed	Mech	Manual	Mixed	Mech
Households using manual emptying (count)	98	10	18	47	0	5	51	10	13
Manual emptying interval									
Households emptying every month (count)	0	0	0	0	0	0	0	0	0
Households emptying a few times per year (count)	24	4	10	5	0	2	19	4	8
Households emptying once per year (count)	28	2	4	13	0	1	15	2	3
Households emptying every few years (count)	46	4	4	29	0	2	17	4	2
Weight average manual emptying interval (years)	1.54	1.33	0.96	1.85	-	1.33	1.25	1.33	0.82
Households sharing									
Households sharing (count)	49	6	13	21	0	3	28	6	10
Households not-sharing (count)	39	3	4	21	0	0	18	3	4
Proportion of manual emptying households sharing	56%	67%	76%	50%	-	100%	61%	67%	71%

Modelling scenario on household emptying volume and interval

Figure S22 shows the full results from the modelling scenario on Total Annualised Cost per Household (TACH) and Total Annualised Volumetric Cost (TAVC) based on variations in household emptying volumes and interval.

Figure S22a shows that about 50% of households report emptying volumes able to be transported by a tricycle in a single trip (i.e. up to six barrels) and that fewer than 5% report emptying volumes requiring multiple trips by a pickup truck (i.e. greater than 14 barrels).

Figure S22b shows a clear relationship between emptying interval and TACH: households reporting lower emptying intervals have a higher TACH but this does not account of differences in emptying volumes which are directly linked to the largest cost voice, emptiers' wages, which are modelled as being paid per barrel.

Figure S22c compares annualised sludge emptying rate (which consolidates emptying volume and emptying interval) with TACH and shows a strong positive linear relationship.

Figure S22d and Figure S22e compare TAVC and TACH with emptying interval between the two cities, showing some difference between Blantyre and Kampala.

Figure S22f shows that this is largely explained by differences in the annualised sludge emptying rate, where there is much more range in Blantyre than in Kampala.

Figure S22g plots the same data as Figure S22c but the series are city rather than emptying interval, and shows that after adjusting for annualised sludge emptying rate, the difference between the two cities is not observable.

Figure S22k shows a weak relationship between the annualised sludge emptying rate and TAVC which highlights the variation in TAVC, irrespective of the annualised sludge emptying rate.

Figure S22l shows this variation by comparing TAVC and the emptying volume per job, showing that TAVC is effectively minimised when a single pickup truck is fully utilised (14 barrels) and that low volume emptying has an exponentially higher TAVC.

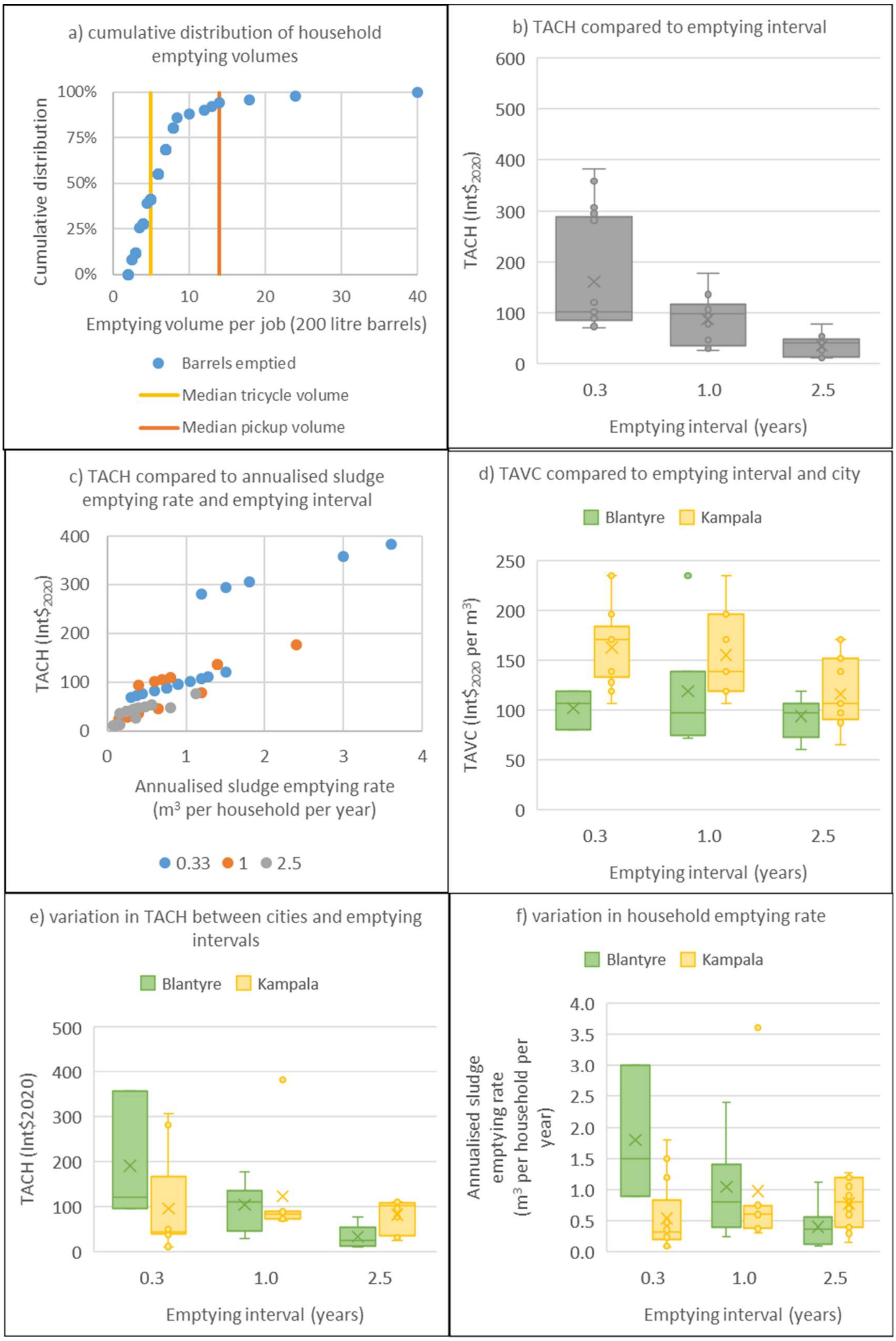
a) cumulative distribution of household emptying volumes
Cumulative distribution
100%
75%
50%
25%
0%
0
10
20
30
40
Emptying volume per job (200 litre barrels)
Barrels emptied
Median tricycle volume
Median pickup volume
b) TACH compared to emptying interval
TACH (Int$2020)
600
500
400
300
200
100
0
0.3
1.0
2.5
Emptying interval (years)
c) TACH compared to annualised sludge emptying rate and emptying interval
TACH (Int$2020)
400
300
200
100
0
0
1
2
3
4
Annualised sludge emptying rate
(m³ per household per year)
0.33
1
2.5
d) TAVC compared to emptying interval and city
Blantyre
Kampala
TAVC (Int$2020 per m³)
250
200
150
100
50
0
0.3
1.0
2.5
Emptying interval (years)
e) variation in TACH between cities and emptying intervals
Blantyre
Kampala
TACH (Int$2020)
500
400
300
200
100
0
0.3
1.0
2.5
Emptying interval (years)
f) variation in household emptying rate
Blantyre
Kampala
Annualised sludge emptying rate
(m³ per household per year)
4.0
3.5
3.0
2.5
2.0
1.5
1.0
0.5
0.0
0.3
1.0
2.5
Emptying interval (years)

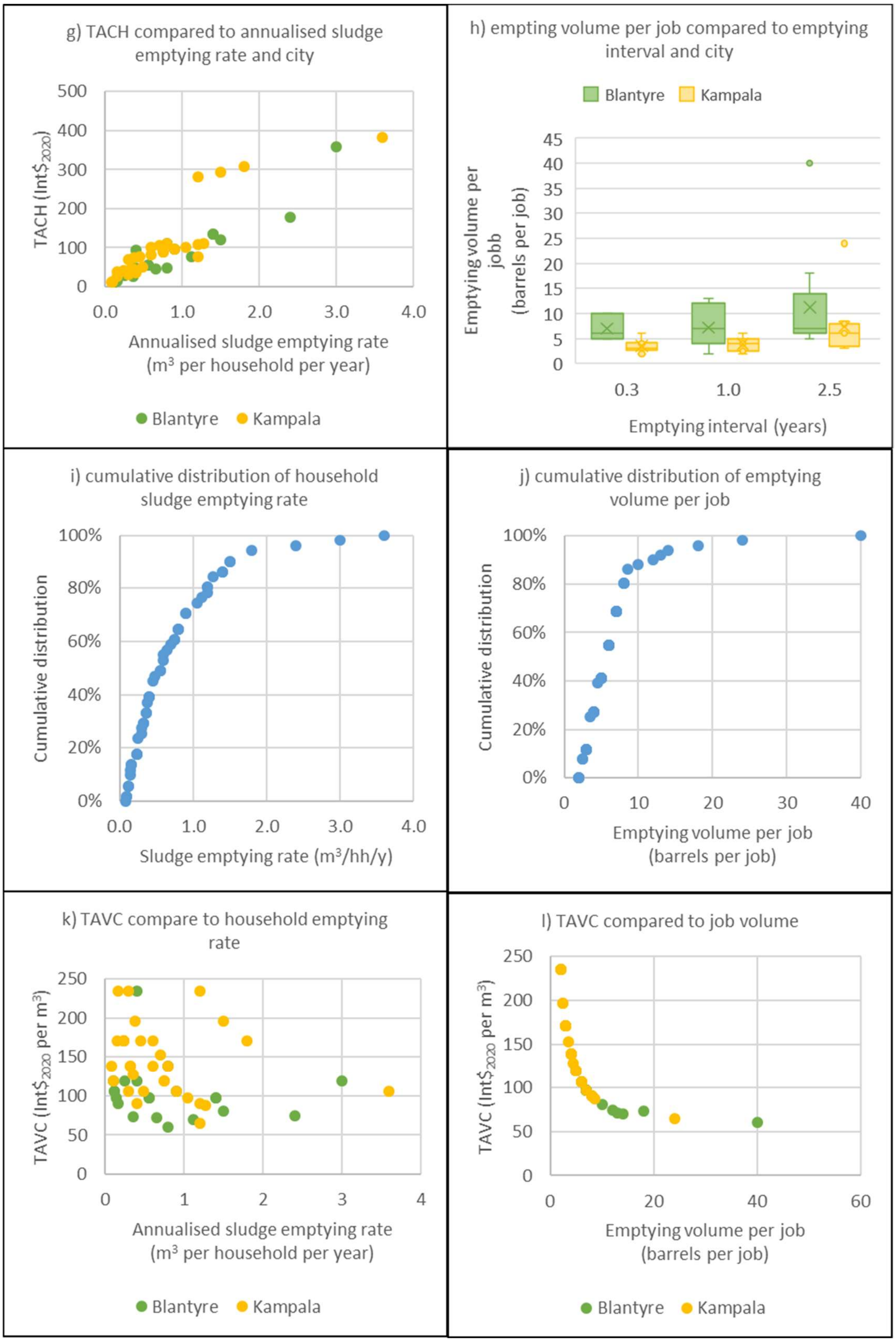

Figure S22 Total Annualised Cost per Household (TACH) and Total Annualised Volumetric Cost (TAVC) based on household (hh) emptying characteristics: interval and volume. Barrels emptied normalised to 200 litres.
